# Water intake, hydration status and 2-year changes in cognitive performance: a prospective cohort study

**DOI:** 10.1186/s12916-023-02771-4

**Published:** 2023-03-08

**Authors:** Stephanie K. Nishi, Nancy Babio, Indira Paz-Graniel, Lluís Serra-Majem, Jesús Vioque, Montserrat Fitó, Dolores Corella, Xavier Pintó, Aurora Bueno-Cavanillas, Josep A. Tur, Laura Diez-Ricote, J. Alfredo Martinez, Carlos Gómez-Martínez, Andrés González-Botella, Olga Castañer, Andrea Alvarez-Sala, Cristina Montesdeoca-Mendoza, Marta Fanlo-Maresma, Naomi Cano-Ibáñez, Cristina Bouzas, Lidia Daimiel, María Ángeles Zulet, John L. Sievenpiper, Kelly L. Rodriguez, Zenaida Vázquez-Ruiz, Jordi Salas-Salvadó

**Affiliations:** 1 Universitat Rovira i Virgili, Departament de Bioquímica i Biotecnologia, Unitat de Nutrició, Reus, Spain; 2 Institut d’Investigació Sanitària Pere Virgili (IISPV), Reus, Spain; 3 Centro de Investigación Biomédica en Red Fisiopatología de la Obesidad y la Nutrición (CIBEROBN), Institute of Health Carlos III, Madrid, Spain; 4 Toronto 3D (Diet, Digestive Tract and Disease) Knowledge Synthesis and Clinical Trials Unit, Toronto, ON Canada; 5 Clinical Nutrition and Risk Factor Modification Centre, St. Michael’s Hospital, Unity Health Toronto, Toronto, ON Canada; 6 Research Institute of Biomedical and Health Sciences (IUIBS), University of Las Palmas de Gran Canaria & Centro Hospitalario Universitario Insular Materno Infantil (CHUIMI), Canarian Health Service, Las Palmas de Gran Canaria, Spain; 7 CIBER de Epidemiología y Salud Pública (CIBERESP), Instituto de Salud Carlos III (ISCIII), 28029 Madrid, Spain; 8 Instituto de Investigación Sanitaria y Biomédica de Alicante. Universidad Miguel Hernández (ISABIAL-UMH), Alicante, Spain; 9 Unit of Cardiovascular Risk and Nutrition, Institut Hospital del Mar de Investigaciones Médicas Municipal d’Investigació Médica (IMIM), Barcelona, Spain; 10 Department of Preventive Medicine, University of Valencia, Valencia, Spain; 11 Lipids and Vascular Risk Unit, Internal Medicine, Hospital Universitario de Bellvitge-IDIBELL, Hospitalet de Llobregat, Barcelona, Spain; 12 School of Medicine, Universitat de Barcelona, 08907 Barcelona, Spain; 13 Department of Preventive Medicine and Public Health, University of Granada, Granada, Spain; 14 Research Group on Community Nutrition & Oxidative Stress, University of Balearic Islands, 07122 Palma de Mallorca, Spain; 15 Nutritional Control of the Epigenome Group, Precision Nutrition and Obesity Program, IMDEA Food, CEI UAM + CSIC, 28049 Madrid, Spain; 16 Department of Nutrition, Food Sciences, and Physiology, Center for Nutrition Research, University of Navarra, IdiSNA, Pamplona, Spain; 17 Precision Nutrition and Cardiometabolic Health Program, IMDEA Food, CEI UAM + CSIC, Madrid, Spain; 18 Centro de Salud Raval de Elche, Alicante, Spain; 19 Instituto de Investigación Biosanitaria Granada, IBS-Granada, Granada, Spain; 20 Departamento de Ciencias Farmacéuticas y de la Salud, Facultad de Farmacia, Universidad San Pablo-CEU, CEU Universities, Urbanización Montepríncipe, Boadilla del Monte, 28660 Spain; 21 Department of Nutritional Sciences, Faculty of Medicine, University of Toronto, Toronto, ON Canada; 22 Department of Medicine, University of Toronto, Toronto, ON Canada; 23 Division of Endocrinology & Metabolism, St. Michael’s Hospital, Toronto, ON Canada; 24 Li Ka Shing Knowledge Institute, St. Michael’s Hospital, Toronto, ON Canada; 25 Departament of Occupational Risk Prevention, Virgen de la Arrixaca’s Hospital (HCUVA), Murcia, Spain; 26 Department of Preventive Medicine and Public Health, Instituto de Investigación Sanitaria de Navarra (IdiSNA), University of Navarra, Pamplona, Spain

**Keywords:** Hydration, Water, Fluids, Serum osmolarity, Serum osmolality, Cognition, Cognitive function, Cognitive performance, PREDIMED-plus

## Abstract

**Background:**

Water intake and hydration status have been suggested to impact cognition; however, longitudinal evidence is limited and often inconsistent. This study aimed to longitudinally assess the association between hydration status and water intake based on current recommendations, with changes in cognition in an older Spanish population at high cardiovascular disease risk.

**Methods:**

A prospective analysis was conducted of a cohort of 1957 adults (aged 55–75) with overweight/obesity (BMI between ≥ 27 and < 40 kg/m^2^) and metabolic syndrome from the PREDIMED-Plus study. Participants had completed bloodwork and validated, semiquantitative beverage and food frequency questionnaires at baseline, as well as an extensive neuropsychological battery of 8 validated tests at baseline and 2 years of follow-up. Hydration status was determined by serum osmolarity calculation and categorized as < 295 mmol/L (hydrated), 295–299.9 mmol/L (impending dehydration), and ≥ 300 mmol/L (dehydrated). Water intake was assessed as total drinking water intake and total water intake from food and beverages and according to EFSA recommendations. Global cognitive function was determined as a composite *z*-score summarizing individual participant results from all neuropsychological tests. Multivariable linear regression models were fitted to assess the associations between baseline hydration status and fluid intake, continuously and categorically, with 2-year changes in cognitive performance.

**Results:**

The mean baseline daily total water intake was 2871 ± 676 mL/day (2889 ± 677 mL/day in men; 2854 ± 674 mL/day in women), and 80.2% of participants met the ESFA reference values for an adequate intake. Serum osmolarity (mean 298 ± 24 mmol/L, range 263 to 347 mmol/L) indicated that 56% of participants were physiologically dehydrated. Lower physiological hydration status (i.e., greater serum osmolarity) was associated with a greater decline in global cognitive function *z*-score over a 2-year period (*β*: − 0.010; 95% CI − 0.017 to − 0.004, *p*-value = 0.002). No significant associations were observed between water intake from beverages and/or foods with 2-year changes in global cognitive function.

**Conclusions:**

Reduced physiological hydration status was associated with greater reductions in global cognitive function over a 2-year period in older adults with metabolic syndrome and overweight or obesity. Future research assessing the impact of hydration on cognitive performance over a longer duration is needed.

**Trial registration:**

International Standard Randomized Controlled Trial Registry, ISRCTN89898870. Retrospectively registered on 24 July 2014

**Supplementary Information:**

The online version contains supplementary material available at 10.1186/s12916-023-02771-4.

## Background

Cognitive decline is an important public health concern given 55 million people have been diagnosed with dementia and almost 80 million people are projected to be affected by 2030 [1]. Cognitive function is particularly important, especially as the population ages, because it determines the maintenance of our independence, the performance of everyday activities, and the quality of life [1]. Cognitive decline and dementia have a diverse etiology, and effective treatment is still not available [2]. For this reason, prevention strategies targeting modifiable factors, such as nutritional habits and dietary intake to slow the development of cognitive impairment, remain a promising public health approach [2]. Evidence to date assessing individual dietary factors and dietary patterns in relation to cognitive health has been summarized in the Lancet Neurology [3], while there is information related to various macro- and micronutrients and patterns, evidence related to water appears limited.

Water intake is a nutritional habit that is often overlooked, yet it is considered essential for the optimal physiological function of the human body [4]. As the most abundant component of the human body, water intake and optimal hydration are considered key aspects for the proper functioning of organ systems, aiding, among others, in efficient digestion, elimination of toxins, energy production, thermoregulation, and joint lubrication, as well as a multitude of biochemical reactions [5, 6]. Furthermore, proper hydration is thought to be important for optimal cognitive functioning as it plays a vital role in neural conductivity [7].

Dehydration occurs when the body loses more water than is taken in, whereas hypohydration refers to the state of water deficit; these conditions result in less-than-optimal hydration and may elicit adverse physiological consequences [8]. For the purposes of this paper, dehydration will be the term used to encompass the state of improper hydration due to unbalanced water loss or water deficit. In Europe, the percentage of the population reported to have inadequate water intake is estimated to vary from 5 to 35% [9–11]. Understanding water intake and hydration status is of particular importance in older adults as this population tends to be less likely to meet recommendations on water intake and is at greater risk for dehydration due to blunted sensitivity to thirst signals, lower body reserves due to reduced muscle mass, reduced ability to deal with heat stress, and use of medications or laxatives with diuretic effects [12–15]. Multiple health organizations and dietary guidelines have acknowledged that adequate hydration status is associated with the preservation of physical and mental functions and that water intake is the best way to achieve hydration [16–19].

To maintain adequate hydration status, several countries and public organizations have proposed water intake recommendations for the public [20, 21]. These recommendations have been determined based on data from population studies and accounted for water from beverage and food sources [22]. For instance, the European Food Safety Authority (EFSA), based on data from population studies from 13 European countries, proposed the dietary reference values (DRV) for the adequate intake (AI) of water which increases with age up to 2.5 L and 2.0 L of water daily for men and women (aged 14 to 70 years), respectively [20]. Similarly, the Institute of Medicine (IOM) proposed increasing water intake levels with age up to 3.7 L/day and 2.7 L/day (aged 19 to 70 years) in men and women, respectively. Yet, the World Health Organization and other guidelines related to cognitive health do not currently include recommendations related to water intake or hydration status [23–25]. Further understanding of health behaviors, such as cognitive functioning, related to something as fundamental as water intake can have a substantial impact on public health.

Inadequate water intake and dehydration have been associated with existing signs of cognitive impairment among older adults living in long-term care facilities [26–29]. Moreover, acute studies have shown that dehydration and water supplementation affect mood and cognitive performance [30]. However, fluid and water intake has received limited attention in epidemiological studies, and the literature scarcely examines water intake as a predictor of cognitive performance among older adults. The few studies that have assessed hydration status as a potential predictor of cognitive function among community-dwelling older adults have been inconclusive [31–34]. Furthermore, to date, few studies have prospectively captured and examined the impact of water and hydration status on cognitive function over a multi-year period. Therefore, the objective of the present analyses was to prospectively investigate the relation between hydration status, water intake, and 2-year changes in cognitive performance in community-dwelling older adults with metabolic syndrome and overweight or obesity.

## Methods

### Study design

This prospective cohort study is based on data collected during the first 2 years of the PREDIMED-Plus (PREvención con DIeta MEDiterránea Plus) study. Briefly, the PREDIMED-Plus study is an ongoing randomized, parallel-group, 6-year multicenter, controlled trial designed to assess the effect of lifestyle interventions on the primary prevention of cardiovascular disease. The primary aim of the trial is to assess the effects of an intensive weight loss intervention based on an energy-reduced Mediterranean diet (MedDiet), physical activity promotion, and behavioral support (intervention group) compared to usual care and dietary counseling only with an energy-unrestricted MedDiet (control group) on the prevention of cardiovascular events. Details of the design and methods of PREDIMED-Plus have been previously described [35, 36] and are available at https://www.predimedplus.com/.

### Ethics, consent, and permissions

The PREDIMED-Plus study protocol and procedures were approved by the Research Ethics Committees from each of the participating centers, and the study was registered with the International Standard Randomized Controlled Trial Registry (ISRCTN; ISRCTN89898870). All participants provided written informed consent.

### Study participants

PREDIMED-Plus participants were recruited from 23 centers across Spain between September 2013 and December 2016. A total of 6874 adults met the eligibility criteria and were randomly allocated in a 1:1 ratio to either the intervention or the control group. Couples sharing the same household were randomized together, and the couple was used as a unit of randomization. Eligible participants were community-dwelling adults (aged 55 to 75 for men; 60 to 75 for women) with overweight or obesity (BMI: 27 to 40 kg/m^2^) who met at least three criteria for metabolic syndrome (35), without previous cardiovascular events or diagnosed neurodegenerative diseases at baseline. All participants provided written informed consent.

The present longitudinal analysis involves a sub-study conducted in 10 of the 23 PREDIMED-Plus recruiting centers. Of the participants in the PREDIMED-Plus sub-study who had a completed validated 32-item Spanish fluid intake questionnaire, participants were excluded if they did not have a completed baseline Food Frequency Questionnaire (FFQ) or who reported implausible total energy intakes based on those proposed by Willet (≤ 500 and ≤ 3500 kcal/day in women and ≤ 800 and ≤ 4000 kcal/day in men) [37]. For the water and fluid intake analyses, using the interquartile range method (using a 1.5 multiplier for the first and third quartiles), participants with extreme intakes of fluid (daily fluid intakes for men < 188 mL or > 3862 mL and women < 263 mL or > 3539 mL) were excluded for the assessment of water and fluid intakes. Similarly, participants without blood sample values for urea, sodium, potassium, glucose, and serum osmolarity values < 100 mmol/L were also excluded from the analyses of hydration status and cognition. Furthermore, associations were tested for those participants who had completed the various cognitive tests.

### Assessment of water and fluid intake

A validated, semi-quantitative 32-item Beverage Intake Assessment Questionnaire (BIAQ) [10] and a 143-item validated semi-quantitative FFQ (38) specifying usual portion sizes, were administered by trained dietitians to assess habitual fluid and dietary intakes, respectively. These two questionnaires have been validated within populations of older, Spanish individuals, which are analogous to the current study population, and both have been found to be reproducible with relative validity [10, 38]. The BIAQ recorded the frequency of consumption of various beverage types during the month prior to the visit date. The average daily fluid intake from beverages was estimated from the servings of each type of beverage. The questionnaire items on beverages included: tap water, bottled water, natural fruit juices, bottled fruit juices, natural vegetable juices, bottled vegetable juices, whole milk, semi-skimmed milk, skimmed milk, drinking yogurt, milkshakes, vegetable drinks, soups, jellies and sorbets, soda, light/zero soda, espresso, coffee, tea, beer, non-alcoholic beer, wine, spirits, mixed alcoholic drinks, energy drinks, sports drinks, meal replacement shakes, and other beverages. The water and nutrient contents of the beverages were estimated mainly using the CESNID Food Composition Tables [39], complemented with data from the BEDCA Spanish Database of Food Composition [40].

The FFQ collected data on food intake based on the year prior to the visit according to nine possible frequency categories, which ranged from “never or almost never” to “> 6 portions/day” and based on the dietary guidelines for the Spanish population [41]. The information collected was converted into grams per day, multiplying portion sizes by consumption frequency and dividing the result by the period assessed. Ten food groups composed of vegetables, fruits, legumes, cereals, dairy beverages, meat and poultry, fats, nuts, fish/seafood, and other foods were determined to assess the contribution of foods to total water intake. Food groups and energy intake were estimated using Spanish food composition tables [42, 43]. Drinking water intake, water intake from all fluids, total water intake, EFSA total fluid water intake (TFWI), and EFSA total water intake (TWI) were computed (descriptions summarized in Additional file 1: Table S1). Drinking water intake was estimated based on tap and bottled water intakes based on BIAQ responses. Water intake from all fluids was computed from tap and bottled water, plus water from other beverages based on responses to the BIAQ. Total water intake encompassed water intake from all fluids in addition to water present in food sources based on responses to the FFQ. Water intake was further categorized based on established reference values. The EFSA recommendations for total water intake (EFSA TWI) for older adults (2.5 L/day and 2.0 L/day for men and women, respectively) in conditions of moderate environmental temperature and moderate physical activity [20] were used as reference values. Further categorizations were determined based on total water intake from fluids alone, based on EFSA recommendations (EFSA TFWI), where recommended levels for older adults are set to at least 2.0 L/day and 1.6 L/day for men and women, respectively [20].

### Assessment of hydration status

Hydration status was estimated based on calculated serum osmolarity (SOSM), which is considered a more reliable biomarker of hydration status than urinary markers in older adults [44]. Fasting serum glucose, urea, sodium, and potassium were measured by standard methods. Blood urea nitrogen was determined from urea values using the conversion factor of 0.357 and reported in mmol/L. With all relevant serum analyte measures presented in mmol/L, SOSM was estimated using the following equation [45]:

$$\textrm{SOSM}=1.86\ast \left(\textrm{sodium}+\textrm{potassium}\right)+1.15\ast \textrm{glucose}+\textrm{blood}\ \textrm{urea}\ \textrm{nitrogen}+14$$

Based on this equation, dehydration, impending dehydration, and hydrated statuses were defined as SOSM > 300, 295–300, and < 295 mmol/L, respectively [28, 46].

### Assessment of cognitive performance

A battery of 8 neuropsychological tests assessing different cognitive domains was administered at baseline and 2-year follow-up by trained staff to assess cognitive performance. The following tests were assessed: the Mini-Mental State Examination (MMSE), two Verbal Fluency Tests (VFTs), two Digit Span Tests (DSTs) of the Wechsler Adult Intelligence Scale-III (WAIS-III), the Clock Drawing Test (CDT), and two Trail Making Tests (TMTs).

Briefly, a Spanish-validated version of the MMSE questionnaire, a commonly used cognitive screening test, was used in the present analysis [47]. A higher MMSE score indicates better cognitive performance [48]. Verbal ability and executive function were evaluated using the VFTs, which consist of two parts: the semantic verbal fluency task-animal category version (VFT-a) and the phonemic verbal fluency task-letter “p” version (VFT-p) [49]. The DST of the WAIS-III Spanish version assessed attention and memory. The DST Forward Recall (DST-f) is representative of attention and short-term memory capacity, and the DST Backward Recall (DST-b) is considered as a test of working memory capacity [50, 51]. The CDT-validated Spanish version was mainly used to evaluate visuospatial and visuo-constructive capacity [52–54]. Lastly, the TMT, another tool often used to assess executive function, consists of two parts. Part A (TMT-A) assessed attention and processing speed capacities, and part B (TMT-B) further examined cognitive flexibility [55]. All instruments included in the cognitive battery have been standardized for the Spanish population in the age range of the study population.

To assess overall cognitive function, a global cognitive function (GCF) score was determined as the main outcome measure, in addition to evaluating the individual neuropsychological tests (supplementary analyses). Raw scores at baseline and scores of changes at 2 years of follow-up for each individual cognitive assessment, as well as GCF, were standardized using the mean and standard deviation from the baseline measurements as normative data, creating *z*-scores [56]. GCF was calculated as a composite *z*-score of all 8 assessments, adding or subtracting each individual test value based on whether a higher score indicates higher or lower cognitive performance, respectively, using the formula:

$$\textrm{GCF}=\left({\textrm{Z}}_{\textrm{MMSE}}+{\textrm{Z}}_{\textrm{CDT}}+{\textrm{Z}}_{\textrm{VFT}-\textrm{a}}+{\textrm{Z}}_{\textrm{VFT}-\textrm{p}}+\left(-{\textrm{Z}}_{\textrm{TMT}-\textrm{A}}\right)+\left(-{\textrm{Z}}_{\textrm{TMT}-\textrm{B}}\right)+{\textrm{Z}}_{\textrm{DST}-\textrm{f}}+{\textrm{Z}}_{\textrm{DST}-\textrm{b}}\right)/8$$

### Covariate assessment

The trained staff collected baseline socio-demographic (i.e., sex, age, education level, and civil status) and lifestyle (i.e., physical activity, total energy intake, alcohol intake, caffeine consumption [57], sleeping habits, and smoking status) related variables, as well as information about medication use, in face-to-face interviews using self-reported general questionnaires and a 143-item validated semi-quantitative FFQ for the dietary related variables [38], which were further estimated using Spanish food composition tables [42, 43]. Leisure time physical activity was estimated using the validated Minnesota-REGICOR Short Physical Activity questionnaire [58]. These socio-demographic and lifestyle variables were considered as possible covariates because of reports that younger adults, women, individuals with higher educational attainment, married, more active, greater consumers, and non-smokers tend to consume higher amounts of fluids from beverages and foods and hence more likely to meet recommendations on water intake [59, 60]. Alcohol was accounted for as a potential covariate as it may act as a diuretic at certain levels [61] as well as being associated with an elevated risk of dementia when consumed regularly [62]. Similarly, caffeinated beverages may have a mild diuretic effect [63], as well as may affect attention and alertness [64] and could be associated with reduced cognitive decline and dementia risk [65]. Sleeping habits have also been associated with cognitive health [66]. Anthropometric measures, including weight and height were measured by trained staff using calibrated scales and wall-mounted stadiometers, respectively. Body mass index (BMI), which may modify the relationship between water intake and hydration status [67], was calculated as weight in kilograms divided by height in meters squared. History of chronic disease (i.e., type 2 diabetes, hypertension, and hypercholesterolemia) was self-reported or collected from patient medical records and included as these conditions may cause fluid imbalance, cause dehydration, and have been associated with cognitive performance possibly leading to mild cognitive impairment [68–70]. Depressive symptomatology was evaluated using the Beck Depression Inventory-II (BDI-II), given the association observed with cognitive health [71] where depressive symptomatology risk was established as a score ≥ 14 [72].

### Statistical analyses

For the present analyses, a prospective cohort study was conducted within the framework of the PREDIMED-Plus study using the database updated to December 22, 2020. Participants were categorized into quantiles based on baseline water intake (drinking water, water intake from all fluids, total water intake), recommended categories of water intake (EFSA TWI, EFSA, TFWI), and hydration status according to serum osmolarity. Baseline characteristics of participants for each category and quantile of water intake and hydration status were presented as numbers and percentages using Pearson’s chi-square test for categorical variables and means ± standard deviations or median (interquartile range [P25–P75]) using one-way ANOVA or Kruskal Wallis test for continuous variables, as appropriate.

Multivariable linear regression models were fitted to assess longitudinal associations comparing the 2-year change in cognitive function across baseline variables of hydration status and water intake and for meeting the EFSA recommendations for TWI and TFWI [20]. When analyses were performed with categorical variables, *p* for trend was calculated. The *p* for linear trend was calculated by assigning the median value of each category and modeling it as a continuous variable.

Multivariable linear regression models were adjusted for several potential confounders. Model 1 adjusted for age (years), sex (man or woman), intervention group, participating center size (< 100, 100 to < 150, 150 to < 200, ≥ 200 participants for hydration status and < 100, 100 to < 200, 200 to < 300, ≥ 300 participants for fluid-related analyses), respective baseline cognitive function score, and corrected for clusters (to account for couples living in the same household being randomized as a single unit). Model 2 was additionally adjusted for BMI (kg/m^2^), educational level (primary, secondary, or college), civil status (single, divorced or separated, married, widower), smoking status (current, former, or never), and physical activity (METs/min/day). Model 3 was additionally adjusted for sleeping habits (hours of nighttime sleep), depressive symptomatology (yes/no), diabetes prevalence (yes/no), hypertension (yes/no), hypercholesterolemia (yes/no), total energy intake (kJ per day), alcohol consumption in g/day (and adding the quadratic term), and caffeine intake (g/day). To assess the linear trend, the median value of each category of exposure variables (hydration status and various assessments of water and fluid intake) was assigned to each participant and was modeled as continuous variables in linear regression models. The Bonferroni correction was used to correct for multiple comparisons and reduce the risk of a type 1 error.

Several stratified and sensitivity analyses were additionally performed to test the robustness of the findings. First, sex-stratified regression approaches were employed to examine the relationships between hydration status and these water and fluid intake categories and 2-year changes in global cognitive function. Sensitivity analyses were additionally performed, testing the addition of estimated glomerular filtration rate (eGFR), an indicator of renal function derived based on serum creatinine level, age, and sex [73], or dietary intake covariates (amount [g/day] of vegetables, fruits, legumes, grains, non-fluid dairy, meat, oils, fish, nuts, and pastries determined via the validated 143-item semi-quantitative FFQ [19]) to the multivariable models, but also after removal of participants with baseline MMSE < 24 (mild dementia and poorer) [74], or the removal of participants with extreme GCF *z*-scores at baseline (< 5% and > 95%).

The data were analyzed using the Stata 14 software program (StataCorp LP, TX, USA), and the results were originally considered statistically significant at a *p*-value (2-tailed) < 0.05, and after the Bonferroni correction, statistical significance was considered at a *p*-value (2-tailed) < 0.005.

## Results

A total of 1957 participants (mean age 65.0 ± 4.9 years and 50.5% women) were available for the assessment of water and fluid intake and 1192 participants for the assessment of hydration status after excluding missing values or implausible data (Additional file 1: Fig. S1). Table 1 presents the baseline characteristics of the study population according to sex, water (tap and bottle) intake amount, and hydration status. The median (range) consumption of drinking water intake in men and women was 900 (0 to 3100) and 900 (0 to 2700) mL/day, respectively. Compared with participants in the group with the lowest drinking water intake (< 500 mL/day), those with the highest drinking water intake (1.8 to 3.1 L/day) were more likely to be younger (*p* < 0.001), have a higher BMI (*p* = 0.001), and have a lower alcohol intake (*p* < 0.001). Compared to participants considered to be hydrated according to serum osmolarity status, participants considered to be dehydrated tended to be older (*p* = 0.008), women (*p* = 0.008), have type 2 diabetes (*p* < 0.001), have depressive symptoms (*p* = 0.025), and less likely to drink alcohol (*p* = 0.002). Participants involved in the present analyses did not differ from the rest of the participants enrolled in the PREDIMED-Plus trial in terms of age, sex, BMI, and prevalence of obesity and type 2 diabetes (*p* > 0.05 for all comparisons). Furthermore, 80% of participants (69% of men and 90% of women) met the EFSA fluid intake recommendations based on questionnaire responses, yet serum osmolarity values indicated over 50% of participants were physiologically dehydrated with only 10% of participants being considered physiologically hydrated based on serum osmolarity levels.

**Table 1:** Baseline characteristics of the participants according to sex, categories of water intake, and hydration status

Trait	Total	Women	Men	***p***-value	Categories of water intake					Categories of estimated SOSM^**a**^			
					Lowest	Low-moderate	High-moderate	Highest	***p***-value	Hydrated^**b**^	Impending dehydrated	Dehydrated	***p***-value
Median	900	900	900		400 ml/day	900 ml/day	1300 ml/day	1800 ml/day		292 mmol/L	298 mmol/L	304 mmol/L	
Range	(0–3100)	(0–2700)	(0–3100)		(0–500)	(800–1000)	(1071–1400)	(1800–3100)		(263–294.9)	(295–299.9)	(300–346.5)	
Frequency, *n*	1957	989	968		390	744	546	277		123	400	669	
*Socio-demographic data*													
Age (years)	65. 0± 4.9	66.2 ± 4.0	63.70 ± 5.36	< 0.001	65.0 ± 4.9	65.4 ± 4.8	64.9 ± 5.0	64.0 ± 4.7	**< 0.001**	64.2 ± 5.0	64.3 ± 5.0	65.2 ± 4.8	**0.008**
Sex (women)	989 (50.5)	989 (50.5)	968 (49.5)		188 (48.2)	383 (51.5)	280 (51.3)	138 (49.8)	0.731	47 (38.2)	190 (47.5)	353 (52.8)	**0.008**
Education level													
Primary school	1023 (52.3)	629 (63.6)	394 (40.7)		188 (48.2)	399 (53.6)	297 (54.4)	139 (50.2)		68 (55.3)	211 (52.8)	372 (55.6)	
High school	545 (27.9)	235 (23.8)	310 (32.0)		111 (28.5)	201 (27.0)	148 (27.1)	85 (30.7)		29 (25.6)	112 (28.0)	184 (27.5)	
College	389 (19.9)	125 (12.6)	264 (27.3)	< 0.001	91 (23.3)	144 (19.4)	101 (18.5)	53 (19.1)	0.381	26 (21.1)	77 (19.3)	113 (16.9)	0.636
Civil status													
Single, divorced or separated	242 (12.4)	136 (13.8)	106 (11.0)		47 (12.1)	96 (12.9)	62 (11.4)	37 (13.4)		17 (13.8)	46 (11.5)	77 (11.5)	
Married	1513 (77.3)	684 (69.2)	829 (85.6)		300 (76.9)	560 (75.3)	423 (79.1)	221 (79.8)		97 (78.9)	319 (79.8)	520 (77.7)	
Widower	202 (10.3)	169 (17.1)	33 (3.4)	< 0.001	43 (11.0)	88 (11.8)	52 (9.5)	19 (6.9)	0.311	9 (7.3)	35 (8.8)	72 (10.8)	0.645
*Lifestyle factors*													
Body mass index, kg/m^2^	32.6 ± 3.5	33.0 ± 3.6	32.3 ± 3.4	< 0.001	32.3 ± 3.4	32.7 ± 3.5	32.5 ± 3.5	33.3 ± 3.5	**0.001**	32.3 ± 3.1	32.5 ± 3.4	32.8 ± 3.5	0.245
Physical activity (MET min/day)	348.7 ± 334.4	284.0 ± 244.6	414.8 ± 395.5	< 0.001	330.5 ± 322.4	345.2 ± 335.4	364.1 ± 348.9	352.6 ± 325.6	0.502	423.9 ± 338.2	364.5 ± 337.9	344.2 ± 346.8	0.057
Smoking status													
Never smoked	904 (46.2)	704 (71.2)	200 (20.7)		182 (46.7)	334 (44.9)	263 (48.17)	125 (45.1)		47 (38.2)	175 (43.8)	333 (49.8)	
Former smoker	817 (41.8)	212 (21.4)	605 (62.5)		163 (41.8)	305 (41.0)	222 (40.7)	127 (45.9)		56 (45.5)	177 (44.3)	269 (40.2)	
Current smoker	236 (12.1)	73 (7.4)	163 (16.8)	< 0.001	45 (11.5)	105 (14.1)	61 (11.2)	25 (9.0)	0.729	20 (16.3)	48 (12.0)	67 (10.0)	0.057
Sleeping status, weekdays													
≤ 6 h	697 (35.6)	381 (38.5)	316 (32.6)		141 (36.2)	264 (35.5)	191 (35.0)	101 (36.5)		45 (36.6)	140 (35.0)	237 (35.4)	
6 to ≤ 8 h	1096 (56.0)	517 (52.3)	579 (59.8)		215 (55.1)	412 (55.4)	315 (57.7)	154 (55.6)		65 (52.9)	227 (56.8)	366 (54.7)	
> 8 h	164 (8.4)	91 (9.2)	73 (7.5)	0.003	34 (8.7)	68 (9.1)	40 (7.3)	22 (7.9)	0.929	13 (10.6)	33 (8.3)	66 (9.9)	0.863
Sleeping status, weekends													
≤ 6 h	600 (30.7)	344 (34.9)	256 (26.5)		128 (32.9)	217 (29.3)	161 (29.5)	94 (34.2)		35 (28.5)	122 (30.7)	208 (31.1)	
6 to ≤ 8 h	1111 (56.9)	517 (52.4)	594 (61.5)		207 (53.2)	431 (58.1)	320 (58.6)	153 (55.6)		69 (56.1)	233 (58.5)	362 (54.2)	
> 8 h	241 (12.4)	125 (12.7)	116 (12.0)	< 0.001	54 (13.9)	94 (12.7)	65 (11.9)	28 (10.2)	0.437	19 (15.5)	43 (10.8)	98 (14.7)	0.371
*Disease prevalence at recruitment*													
Diabetes	618 (31.6)	283 (28.6)	335 (34.6)	0.004	118 (30.3)	236 (31.7)	179 (32.8)	85 (30.7)	0.851	30 (24.4)	89 (22.3)	241 (36.0)	**< 0.001**
Hypertension	1640 (83.8)	834 (84.3)	806 (83.3)	0.523	326 (83.6)	626 (84.1)	453 (83.0)	235 (84.8)	0.902	96 (78.1)	329 (82.3)	568 (84.9)	0.136
Hypercholesterolemia	1411 (72.1)	761 (77.0)	650 (67.2)	< 0.001	274 (72.1)	544 (73.5)	380 (71.0)	177 (70.8)	0.742	88 (71.5)	292 (73.0)	487 (72.8)	0.950
Depressive symptoms	428 (21.9)	314 (31.8)	114 (11.8)	< 0.001	94 (24.1)	154 (20.7)	116 (21.3)	64 (23.1)	0.551	20 (16.3)	81 (20.3)	171 (25.6)	**0.025**
*Biochemical parameters*													
Serum potassium (mmol/L)	4.3 ± 0.4	4.3 ± 0.4	4.3 ± 0.4	0.355	4.4 ± 0.4	4.3 ± 0.4	4.28 ± 0.37	4.3 ± 0.4	**0.003**	4.2 ± 0.4	4.3 ± 0.4	4.3 ± 0.4	**0.005**
Serum sodium (mmol/L)	139.4 ± 14.9	139.0 ± 17.1	139.9 ± 12.4	0.219	140.1 ± 12.6	139.1 ± 15.7	139.6 ± 14.5	138.8 ± 16.6	0.492	137.6 ± 2.1	140.2 ± 1.4	142.4 ± 2.1	< 0.001
Glucose (mmol/L)	6.3 ± 1.7	6.3± 1.6	6.4 ± 1.8	0.056	6.29 ± 1.56	6.34 ± 1.75	6.27 ± 1.64	6.43 ± 1.95	0.582	5.7 ± 0.9	6.0 ± 1.2	6.7 ± 2.0	< 0.001
Blood urea nitrogen (BUN, mmol/L)	6.6 ± 1.8	6.6 ± 1.7	6.7 ± 1.9	0.791	6.6 ± 1.7	6.7 ± 1.8	6.7 ± 1.9	6.7 ± 1.8	0.980	5.9 ± 1.5	6.1 ± 1.4	7.0 ± 1.9	< 0.001
SOSM (mmol/L)^a^	298.4 ± 24.4	297.6 ± 28.0	299.1 ± 20.2	0.298	299.2 ± 21.6	297.6 ± 26.6	299.3 ± 19.1	296.9 ± 31.1	0.580	292.3 ± 3.6	297.9 ± 1.4	304.1 ± 3.8	< 0.001
*Dietary intake*													
Alcohol intake (g/day)	9.8 ± 13.3	4.0 ± 6.7	15.7 ± 15.6	< 0.001	12.5 ± 16.2	9.8 ± 12.9	8.7 ± 12.2	8.2 ± 11.1	**< 0.001**	13.3 ± 16.5	11.3 ± 15.3	9.1 ± 13.2	**0.002**
Energy intake (kcal/day)	2391.7 ± 541.4	2238.3 ± 493.1	2548.6 ± 543.8	< 0.001	2422.1 ± 559.5	2391.2 ± 530.4	2374.7 ± 549.0	2384.1 ± 30.8	0.611	2400.0 ± 598.1	2342.4 ± 549.3	2365.7 ± 554.4	0.579
Caffeine intake (mg/day)	33.6 ± 31.8	29.6 ± 27.9	37.6 ± 34.9	< 0.001	38.3 ± 35.2	31.6 ± 29.7	32.6 ± 31.3	34.0 ± 32.6	0.007	35.7 ± 35.1	36.9 ± 35.3	35.8 ± 31.9	0.869
Mediterranean diet score (17-point)	8.5 ± 2.6	8.8 ± 2.6	8.1 ± 2.6	< 0.001	8.4 ± 2.6	8.3 ± 2.6	8.6 ± 2.6	8.7 ± 2.6	0.0619	8.7 ± 2.7	8.6 ± 2.6	8.6 ± 2.5	0.889
Drinking water intake (ml/day)	1031.2 ±465.2	1033.1 ± 458.8	1029.3	0.8551	352.9 ± 123.4	892.1 ± 30.1	1300.5 ± 17.3	1829.2 ± 155.7	< 0.001	1036.0 ± 499.2	1034.7 ± 466.6	1044.3 ± 478.8	0.946
Total fluid intake (ml/day)	1842.4 ± 592.0	1797.2 ± 579.7	1888.7 ± 601.1	0.001	1232.1 ± 454.2	1691.2 ± 388.5	2103.5 ± 406.7	2593.5 ± 413.5	< 0.001	1931.1 ± 643.3	1886.5 ± 662.4	1874.4 ± 646.0	0.672
Total water intake (ml/day)	2871.1 ± 676.2	2853.5 ± 674.8	2889.2 ± 677.4	0.2434	2241.2 ± 500.9	2709.8 ± 493.7	3146.0 ± 516.0	3646.0 ± 562.8	< 0.001	2947.2 ± 710.1	2869.6 ± 743.4	2852.4 ± 735.2	0.421
Meets EFSA TWI recommendations	1569 (80.2)	898 (90.8)	671 (69.3)	< 0.001	186 (47.7)	595 (80.0)	512 (93.8)	276 (99.6)	**<0.001**	99 (80.5)	309 (77.3)	521 (77.9)	0.750
*Cognitive function tests*													
GCF^c^	0.04 ± 0.62	− 0.15 ± 0.61	0.22 ± 0.58	< 0.001	0.10 ± 0.58	0.04 ± 0.64	− 0.03 ± 0.66	0.11 ± 0.55	**0.018**	0.02 ± 0.67	0.13 ± 0.56	0.01 ± 0.64	**0.028**
MMSE	28.14 ± 1.94	27.82 ± 2.18	28.46 ± 1.60	< 0.001	28.33 ± 1.84	28.04 ± 1.98	28.02 ± 2.02	28.36 ± 1.74	**0.008**	28.23 ± 1.91	28.20 ± 2.09	28.14 ± 2.04	0.824
CDT	5.81 ± 1.31	5.68 ± 1.38	5.94 ± 1.23	< 0.001	5.83 ± 1.37	5.77 ± 1.31	5.80 ± 1.27	5.86 ± 1.31	0.767	5.75 ± 1.50	5.90 ± 1.27	5.89 ± 1.20	0.481
VFT-a	15.78 ± 4.74	14.75 ± 4.54	16.82 ± 4.71	< 0.001	16.32 ± 4.54	15.61 ± 4.98	15.49 ± 4.73	16.03 ± 4.31	**0.032**	15.77 ± 5.04	15.72 ± 4.58	15.62 ± 4.70	0.913
VFT-p	11.95 ± 4.40	11.31 ± 4.34	12.61 ± 4.36	< 0.001	12.23 ± 4.47	11.93 ± 4.49	11.65 ± 4.41	12.23 ± 3.98	0.145	11.99 ± 4.11	12.19 ± 4.35	11.46 ± 4.32	**0.023**
TMT-A	55.17 ± 31.40	61.62 ± 32.56	48.94 ± 28.88	< 0.001	53.73 ± 28.75	57.04 ± 33.21	55.77 ± 32.55	51.02 ± 27.04	**0.037**	56.72 ± 28.45	54.97 ± 30.33	57.56 ± 36.13	0.474
TMT-B	136.9 ± 78.4	156.6 ± 82.5	116.8 ± 68.3	< 0.001	129.9 ± 75.4	138.0 ± 79.2	142.9 ± 81.4	132.2 ± 72.6	0.06	153.2 ± 92.3	139.6 ± 81.0	146.4 ± 82.4	0.217
DST-f	8.66 ± 2.46	8.13 ± 2.32	9.19 ± 2.48	< 0.001	8.61 ± 2.33	8.74 ± 2.48	8.50 ± 2.57	8.86 ± 2.34	0.23	8.26 ± 2.62	8.74 ± 2.44	8.73 ± 2.53	0.176
DST-b	5.06 ± 2.20	4.53 ± 1.97	5.58 ± 2.28	< 0.001	5.04 ± 2.25	5.15 ± 2.15	4.85 ± 2.20	5.27 ± 2.19	0.049	4.89 ± 2.30	5.11 ± 2.11	5.02 ± 2.16	0.638

Data are *n* (%) or mean ± SD for categorical and quantitative variables, respectively. The exception is the diet score is represented as mean (range)

Chi-squared is used for categorical variables and one-way ANOVA for quantitative variables

SOSM > 300 mmol/L represented a dehydrated state, 295 to 300 mmol/L represented a state of impending dehydration, and < 294 mmol/L represented a hydrated state

*Abbreviations*: *CDT*, Clock Drawing Test; *DASH*, Dietary Approaches to Stop Hypertension; *DST-f*, Digit Span Test Forward; *DST-b*, Digit Span Test Backward; *GCF*, global cognitive function; *MMSE*, Mini-Mental State Examination; *TMT-A*, Trail Making Test Part A; *TMT-B*, Trail Making Test Part B; *VFT-a*, Verbal Fluency tasks semantical; *VFT-p*, Verbal Fluency Tasks Phonological

^a^SOSM represents calculated serum osmolarity and was calculated using the formula SOSM = 1.86 × (Na+ + K+) + 1.15 × glucose + BUN + 14, where all analytes are in mmol/L

^b^Hydrated includes participants considered to be hydrated (SOSM 285 to < 295 mmol/L) and overhydrated (SOSM < 285 mmol/L). These groups were combined due to the small number of participants (*n* = 3) considered to be overhydrated. Assessment with the removal of these participants did not modify the findings

^c^GCF was calculated using the formula GCF= (Z_MMSE_ + Z_CDT_ + Z_VFT-a_ + Z_VFT-p_ + (−Z_TMT-A_) + (−Z_TMT-B_) + Z_DST-f_ + Z_DST-b_)/8

**Fig. 1:**
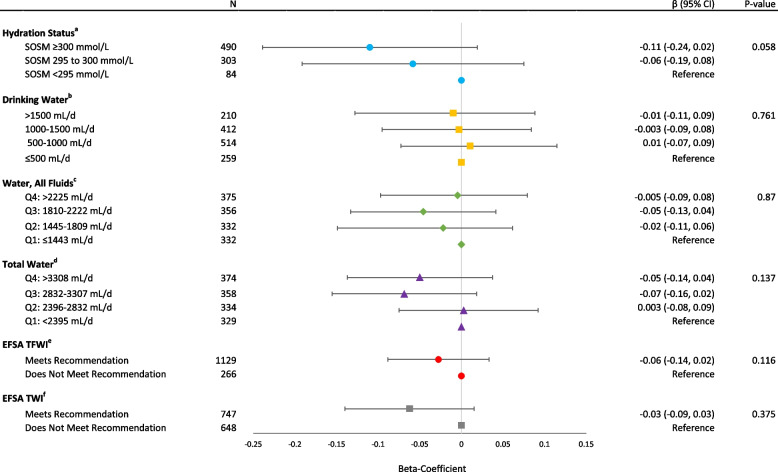
Hydration status, water and fluid intakes categorically with 2-year changes in global cognitive function (*z*-scores). Data are presented as beta-coefficients and 95% CI. Multivariable linear regression models were adjusted for baseline covariates, including baseline GCF score, age (years), sex, intervention PREDIMED-Plus randomized group, and participating center (for hydration status: ≤ 100, 100 to < 150, 150 to < 200, > 200 participants; for fluid-related exposures: ≤ 100, 100 to < 200, 200 to < 300, > 300 participants), body mass index (kg/m^2^), educational level (primary, secondary, or college), civil status (single, divorced or separated, married, widower), smoking habit (current, former, or never), physical activity (METs/min/day), sleep status (hours per day), depressive symptomatology (yes/no), diabetes prevalence (yes/no), hypertension (yes/no), hypercholesterolemia (yes/no), energy intake (kcal/day), alcohol consumption in g/day (and adding the quadratic term), and caffeine intake (mg/day). ^a^Hydration status refers to serum osmolarity, where dehydration, impending dehydration, and hydrated statuses were defined as SOSM > 300, 295–300, and < 295 mmol/L, respectively. ^b^Drinking water refers to tap and bottled water intakes. ^c^Water, all fluids refers to tap and bottled water, plus water from other beverages and fluid food sources, such as soups and smoothies. ^d^Total water refers to water from all fluids in addition to water present in food sources. ^e^EFSA TFWI refers to the recommended levels of total fluid water intake for older adults at 2.0 L/day and 1.6 L/day for men and women, respectively. ^f^EFSA TWI refers to the recommended levels of total water intake, from fluids and foods, for older adults at 2.5 L/day and 2.0 L/day for men and women, respectively. *Abbreviations*: EFSA, European Food Safety Authority; TFWI, total fluid water intake; TWI, total water intake; SOSM, serum osmolarity

**Fig. 2:**
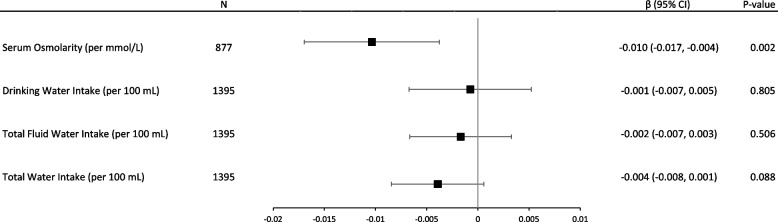
Hydration status, water and fluid intakes continuously with 2-year changes in global cognitive function (z-scores). Data are presented as beta-coefficients and 95% CI. Multivariable linear regression models were adjusted for baseline covariates, including baseline GCF score, age (years), sex, intervention PREDIMED-Plus randomized group, and participating center (≤ 100, 100 to < 150, 150 to < 200, > 200 participants), body mass index (kg/m^2^), educational level (primary, secondary, or college), civil status (single, divorced or separated, married, widower), smoking habit (current, former, or never), physical activity (METs/min/day), sleep status (hours per day), depressive symptomatology (yes/no), diabetes prevalence (yes/no), hypertension (yes/no), hypercholesterolemia (yes/no), energy intake (kcal/day), alcohol consumption in grams/day (and adding the quadratic term), and caffeine intake (mg/day). Beta represents the changes in global cognitive function, expressed as *z*-scores, with each hydration or fluid intake component continuously. Bonferroni correction analyses have been run to correct for multiple comparisons and indicate a *p* < 0.005 is statistically significant

Figures 1 and 2 summarize the multivariable-adjusted *β*-coefficients (95% CIs) of water intake and hydration status categorically and continuously, respectively, with 2-year changes in GCF *z*-scores (full details from all models are presented in Additional file 1: Tables S2-S4). Categorical analyses showed a non-significant trend towards participants considered to have a dehydrated status (*β*: − 0.11; 95% CI: − 0.24 to 0.02; *p* for trend = 0.058) to have a greater decline in global cognitive function compared to those who were considered hydrated (Fig. 1). No significant associations were observed between the various classifications of water intake (i.e., drinking water, all fluids, water from beverage and food sources, and based on ESFA water and fluid recommendations) and 2-year changes in GCF in the multivariable-adjusted models. The results of the continuous linear regression analyses suggest the presence of a significant association between hydration status and global cognitive decline over a 2-year period (*β*: − 0.10; 95% CI: − 0.02 to − 0.004; *p* = 0.002) (Fig. 2).

Additional file 1: Tables S2-S4 show the unadjusted and multivariable-adjusted *β*-coefficients (95% CIs) of water and fluid intake (from both beverage and food sources, assessed individually and combined), as well as hydration status with changes in the global and individual assessments of cognitive function over a 2-year period. When each neuropsychological test was investigated separately, participants with the highest category of intake of drinking water (1.0 to 1.5 L/day) non-statistically significantly presented with a 0.17-point increase (*β*: 0.17; 95% CI: 0.02 to 0.32; *p* for trend = 0.021) in DST-f *z*-score compared to those with the lowest water intake (< 0.5 L/day) over a 2-year period (Additional file 1: Table S2). Total fluid intake showed similar findings where participants in the highest category of intake of total fluid water (2.5 L, range 2.2 to 3.4 L/day) presented with a 0.12-point increase (*β*: 0.12; 95% CI: 0 to 0.24; *p* for trend = 0.041) in DST-f *z*-score compared to those with the lowest water intake (1.1 L, range 0.4 to 1.4 L/day) over a 2-year period (Additional file 1: Table S2). No other associations in the multivariable-adjusted categorical or continuous analyses were observed. Furthermore, post hoc analyses of available data showed no significant differences in changes over time in the various fluid intake assessment variables (over a 2-year duration) or hydration status (over a 1-year duration) (*p* > 0.005) among the participants.

### Stratified analyses

When the analyses were stratified by sex, no changes in significance were observed with the associations between water and fluid intakes, in either categorical or continuous investigations, and global cognitive function. However, when hydration-related analyses were restricted to men or women participants, findings were attenuated when women only were assessed both categorically and continuously (*p* > 0.005), whereas in men those with a SOSM ≥ 300 mmol/L, indicating a dehydrated status, showed a higher cognitive decline over the 2-year period compared to those hydrated (*β*: − 0.20, 95% CI: − 0.36 to − 0.04, *p* = 0.012 and *p*-trend = 0.002), and the significance remained in the continuous analyses (*β*: − 0.013, 95% CI: − 0.022 to − 0.004, *p* = 0.004) (Additional file 1: Figs. S2-S3).

### Sensitivity analyses

Regarding the sensitivity analyses, associations additionally adjusted for eGFR did not significantly modify the findings (Additional file 1: Tables S5-S7). Moreover, associations additionally adjusted for intake of dietary variables, where applicable, including the amount (g/day) of vegetables, fruits, legumes, grains, non-fluid dairy, meat, oils, fish, nuts, and pastries did not significantly modify the findings (Additional file 1: Tables S5-S7). Furthermore, the main results did not substantially change after the removal of extreme GCF baseline *z*-scores (< 5% and > 95%). Whereas following the removal of participants with a baseline MMSE score < 24 (*n* = 51), the categorical association of hydration status with GCF became greater showing a more dehydrated status related to greater 2-year global cognitive decline compared to hydrated participants (*p*-trend = 0.046), and the association of the continuous variables remained significant (*p* = 0.002). The findings related to water and fluid intake did not substantially change with the removal of participants with a baseline MMSE score < 24 (Additional file 1: Tables S5-S7).

## Discussion

To the best of our knowledge, this is the first multi-year prospective cohort study to assess the association between water intake (from fluid and food sources) and hydration status, with subsequent changes in cognitive performance in older Spanish adults with metabolic syndrome and overweight or obesity. In this large sample of older Spanish adults, poorer hydration status was associated with greater global cognitive decline over a 2-year period, particularly in men, whereas water intake, from fluid and/or food sources, and meeting related EFSA recommendations, was not associated with global cognitive function. Nonetheless, when results of cognitive function tests were considered independently, water intake (> 1.5 L/day compared to < 0.5 L/day from tap or bottled water and 2.2 to 4.4 L/day compared to < 1.4 L/day of water from all fluid sources) was related to better attention and short-term memory, as assessed by DST-f, over a 2-year period.

Despite the general acknowledgment that an appropriate level of fluid intake and hydration status is important for health, there have been limited investigations to date assessing the relationship between fluid intake or hydration status and cognitive function. Existing evidence suggests that good hydration status may be associated with better cognitive test results and that mild, induced dehydration can impair cognitive abilities [75], but findings are not consistent and there are only a few studies exploring the relationship of hydration status and hardly any assessing amount of water intake, with cognitive performance in older community-dwelling adults.

Of the few relevant and recent studies that have been conducted, one cross-sectional analysis of 2506 community-dwelling older American adults (aged ≥ 60 years) from the Nutrition and Health Examination Survey (NHANES) 2011–2014 cycles assessed both hydration status and water intake in relation to cognitive performance [34]. In comparison with the present findings, this study found women (but not men) considered to be hydrated, based on a SOSM level between 285 and 289 mmol/L, had better attention and processing speeds, based on a Digit Symbol Substitution test (DSST), than women not at optimal hydration [34]. These cross-sectional findings differ from the present observations where global cognitive function, but not individual tests related to attention and processing, was associated with hydration status. Whereas, correspondingly, water intake (but not hydration status) was positively associated with DST-f, which is similar to the DSST in that it is an indicator of attention as well as short-term memory capacity, and this was seen across all older adults (both men and women). Additionally, in the NHANES study, cognitive test scores were significantly lower among adults who failed to meet EFSA recommendations on adequate intake (AI) of water in bivariate analyses, yet this significance was attenuated in the multivariable analyses among both women and men. Yet, using the alternative AI of daily water intake of 1500 mL or more, which is comparable to the highest drinking water intake group in the present study, women scored higher on the Animal Fluency Test, a measure of verbal fluency and hence executive function, and DSST than women with intake levels below this amount, and findings among men trended in the same direction [34].

Similarly, hydration status has been associated with cognitive function in two cross-sectional studies of older community-dwelling adults by Suhr and colleagues [32, 33]. First, Suhr et al. showed that in 28 healthy community-dwelling older adults (aged 50 to 82 years), a lower hydration status, determined in this study via total body water measured using the bioelectrical impedance method, was related to a decreased psychomotor processing speed, poorer attention, and memory [33]. A second cross-sectional study by Suhr et al. [32] conducted in 21 postmenopausal women (aged 50 to 78 years) reported a positive association between total body water, also measured by the bioelectrical impedance method, and working memory or memory skills.

Moreover, the possible relation between hydration and cognitive function shown by the present findings and demonstrated by the abovementioned cross-sectional studies in older adults align with acute heat- and exercise-induced dehydration studies in younger populations, as illustrated by a meta-analysis of 33 trials (pre-post or crossover design) of 413 adults free of disease (aged < 45 years). It should be noted that these studies assessed dehydration over a period of less than 72 h with 27 (81%) of the studies including only men participants, and 25 (76%) studies involving recreationally or highly athletic individuals. The authors concluded that despite variability among the included studies, dehydration impaired cognitive performance, particularly for tasks involving attention, executive function, and motor coordination when water deficits exceeded 2% body mass loss [76].

Conversely, a cross-sectional study conducted in Poland among 60 community-dwelling older adults (aged 60 to 93 years) found no significant relationship between cognitive performance, as assessed using the MMSE, TMT, and the Babcock Story Recall Test, and hydration status as assessed by urine specific gravity [31]. The discrepancy between the findings from this cross-sectional study and the present PREDIMED-Plus analyses might be because all participants in the cross-sectional study from Poland were considered to be adequately hydrated and hence the authors of that study could not assess the impact of a dehydrated state on cognition.

A noteworthy consideration when interpreting the literature and the main findings of the current study for practical use and in the determination of potential mechanisms of action is the distinction between water intake and water balance (related to hydration status) within the body. When homeostasis of fluids within the body is disrupted, modifying water intake may impact cognitive function, yet due to the dynamic complexity of body water regulation impacting hydration status may be dependent on individualized physiological water intake needs [8]. Thus, while the biological mechanism by which water intake and a hydrated status may reduce the risk of cognitive decline is unclear, evidence suggests that aspects related to hydration and fluid homeostasis or a lack thereof, such as hormone regulation and changes in brain structure, could be a key underlying factor.

Several mechanisms regulate water intake and output to maintain serum osmolarity, and hence hydration status, within a narrow range. Elevated blood osmolarity resulting in the secretion of antidiuretic hormone (ADH), also known as vasopressin or arginine vasopressin, a peptide hormone which acts primarily in the kidneys to increase water reabsorption, is one such mechanism that works to return osmolarity to baseline and preserve fluid balance [77]. In addition to its role in mediating the physiological functions related to water reabsorption and homeostasis, evidence has suggested that ADH participates in cognitive functioning [78] and that the associated cognitive modulations may further interplay with sex hormones [79]. Antidiuretic hormone may be influenced by the androgen sex hormone, which is generally more abundant in the brains of males than in females [80]. As a result, the impact of ADH on cognition could be greater in males [80].

Exercise- and heat-induced acute dehydration studies implicate possible modifications to the brain structure as another potential mechanism of action for an association between water intake, hydration status, and cognitive function. Evidence has proposed that acute dehydration can lead to a reduction in brain volume and subtle regional changes in brain morphology such as ventricular expansion, effects that may be reversed following acute rehydration [81, 82]. Acute dehydration studies have further implicated hydration status in affecting cerebral hemodynamics and metabolism resulting in declines in cerebral blood flow and oxygen supply [83, 84]. A lower vascular and neuronal oxygenation could potentially compromise the cerebral metabolic rate for oxygen, thereby contributing to reductions in cognitive performance [81, 85–88]. Nonetheless, other potential unknown mechanisms cannot be disregarded.

There are several limitations and strengths of the present analyses that need to be acknowledged. The first notable limitation is that the results may not be generalizable to other populations since the participants are older Spanish individuals with metabolic syndrome and overweight or obesity. Second, measurement error and recall bias are possibilities given the use of questionnaires to estimate water and fluid intake and that these rely on responders’ memory which is a component of cognitive function. However, these questionnaires have been validated and determined as reliable methods of assessing long-term intake in the present study population [37, 38]. Third, despite its longitudinal design, water and fluid intake and hydration status were only considered at baseline; however, as the questionnaires measure habitual beverage and food intake, and older adults are considered to have reasonably stable dietary habits [37, 38], this is not expected to significantly impact the findings. Along these lines, the possible effect of seasonality on water intake and osmolarity was not considered a concern in the present analyses as the validation of the fluid questionnaire measurements included assessments at various points throughout the year (baseline vs. 6 months vs 1 year) with no significant differences observed in fluid consumption across the different time points assessed [10]. Hence, the finding of no difference between 6-month intervals, suggests no significant differences between opposing seasons (e.g., winter vs. summer; spring vs. fall). Furthermore, SOSM determination may not necessarily detect acute dehydration or rehydration immediately prior to the cognitive testing, and it is unknown whether observed elevated SOSMs were due to inadequate water intake, ADH abnormality, or other factors. While it is possible that the hydration status of some individuals was misclassified because serum osmolarity was estimated as opposed to being directly measured, the equation has been shown to predict directly measured serum osmolarity well in older adult men and women with and without diabetes or renal issues with a good diagnostic accuracy of dehydration and has been considered a gold standard for the identification of impending and current water-loss dehydration in older adults [44, 45, 89–91]. Lastly, a discrepancy was observed between the percentage of individuals that were considered to have met EFSA fluid intake recommendations and those considered to be dehydrated based on calculated osmolarity. This may have been due to the fact that the EFSA fluid intake recommendations are meant for individuals in good health [20]; whereas the present study population had overweight or obesity, and it has been shown that individuals with higher BMIs have higher water needs related to metabolic rate, body surface area, body weight, and water turnover rates related to higher energy requirements, greater food consumption, and higher metabolic production [92]. Strengths of the present analyses include the longitudinal, prospective design, the large sample size, the use of an extensive cognitive test battery, the use of validated questionnaires, and the robustness of the current findings due to the adjustment of relevant covariates.

## Conclusions

Findings suggest that hydration status, specifically poorer hydration status, may be associated with a greater decline in global cognitive function in older adults with metabolic syndrome and overweight or obesity, particularly in men. Further prospective cohort studies and randomized clinical trials are required to confirm these results and to better understand the link between water and fluid intake, hydration status, and changes in cognitive performance to provide guidance for guidelines and public health.

## Supplementary Information

**Additional file 1: Table S1.** Hydration and water intake definitions. **Table S2.** Associations between cognitive assessments and water and fluid intake exposures. **Table S3.** Associations between cognitive assessments and EFSA fluid intake related guidelines. **Table S4.** Associations between cognitive assessments and hydration status. **Table S5.** Sensitivity analysis in global cognitive function according to water and fluid intake related exposures **Table S6.** Sensitivity analysis in global cognitive function according to EFSA fluid intake related guidelines. **Table S7.** Sensitivity analysis in global cognitive function according to hydration status. **Fig. S1.** Flow diagram of participants. **Fig. S2.** Continuous sensitivity analysis by sex. **Fig. S3.** Categorical sensitivity analysis by sex.

## Data Availability

The dataset supporting the conclusions of this article is available upon request pending application and approval of the PREDIMED-Plus Steering Committee. There are restrictions on the availability of data for the PREDIMED-Plus trial, due to the signed consent agreements around data sharing, which only allow access to external researchers for studies following the project purposes. Requestors wishing to access the PREDIMED-Plus trial data used in this study can make a request to the PREDIMED-Plus trial Steering Committee chair: jordi.salas@urv.cat. The request will then be passed to members of the PREDIMED-Plus Steering Committee for deliberation.

## Abbreviations

ADH: Antidiuretic hormone
AI: Adequate intake
BDI-II: Beck Depression Inventory-II
BEDCA: Base de Datos Española de Composición de Alimentos
BIAQ: Beverage Intake Assessment Questionnaire
BMI: Body mass index
CESNID: El Centro de Enseñanza Superior de Nutrición y Dietética
CDT: Clock Drawing Test
CI: Confidence interval
DRV: Dietary reference value
DSST: Digit Symbol Substitution Test
DST-b: DST Backward Recall
DST-f: DST Forward Recall
EFSA: European Food Safety Authority
eGFR: Estimated glomerular filtration rate
En: Energy
FFQ: Food Frequency Questionnaire
GCF: Global cognitive function
IOM: Institute of Medicine
ISRCTN: International Standard Randomized Controlled Trial Registry
MedDiet: Mediterranean diet
METs: Metabolic equivalents
MMSE: Mini-Mental State Examination
NHANES: Nutrition and Health Examination Survey
PREDIMED: PREvención con DIeta MEDiterránea
Q: Quartile
SOSM: Serum osmolarity
TFWI: Total fluid water intake
TMT-A: Trail Making Test Section A
TMT-B: Trail Making Test Section B
TWI: Total water intake
VFT-a: Verbal Fluency Test Animals Category
VFT-p: Verbal Fluency Test Letter “p” Category
WAIS-III: Wechsler Adult Intelligence Scale-III

## References

[1] Gauthier S, Rosa-Neto P, Morais JA, Webster C. World Alzheimer Report 2021: Journey through the diagnosis of dementia. London: Alzheimer´s Disease International; 2021.

[2] Petersen RC, Lopez O, Armstrong MJ, Getchius TSD, Ganguli M, Gloss D (2018). Practice guideline update summary: mild cognitive impairment report of the guideline development, dissemination, and implementation. Neurology..

[3] Scarmeas N, Anastasiou CA, Yannakoulia M (2018). Nutrition and prevention of cognitive impairment. Lancet Neurol.

[4] Johnson EC, Adams WM (2020). Water intake, body water regulation and health. Nutrients..

[5] Lorenzo I, Serra-Prat M, Carlos YJ (2019). The role of water homeostasis in muscle function and frailty: a review. Nutrients..

[6] Jéquier E, Constant F (2010). Water as an essential nutrient: the physiological basis of hydration. Eur J Clin Nutr.

[7] Kleiner SM (1999). Water. J Am Diet Assoc.

[8] Armstrong L, Johnson E (2018). Water intake, water balance, and the elusive daily water requirement. Nutrients..

[9] Nissensohn M, Sánchez-Villegas A, Ortega RM, Aranceta-Bartrina J, Gil Á, González-Gross M (2016). Beverage consumption habits and association with total water and energy intakes in the Spanish population: findings of the ANIBES study. Nutrients..

[10] Ferreira-Pêgo C, Nissensohn M, Kavouras SA, Babio N, Serra-Majem L, Águila AM (2016). Relative validity and repeatability in a Spanish population with metabolic syndrome from the PREDIMED-PLUS study. Nutrients..

[11] Nissensohn M, Sánchez-Villegas A, Galan P, Turrini A, Arnault N, Mistura L (2017). Beverage consumption habits among the European population: association with total water and energy intakes. Nutrients..

[12] Rolls BJ, Phillips PA (2009). Aging and disturbances of thirst and fluid balance. Nutr Rev.

[13] Kenney WL, Tankersley CG, Newswanger DL, Hyde DE, Puhl SM, Turner NL (1990). Age and hypohydration independently influence the peripheral vascular response to heat stress. J Appl Physiol.

[14] Begg DP (2017). Disturbances of thirst and fluid balance associated with aging. Physiol Behav.

[15] Hooper L, Bunn D, Jimoh FO, Fairweather-Tait SJ (2014). Water-loss dehydration and aging. Mech Ageing Dev.

[16] Lieberman HR (2007). Hydration and cognition: a critical review and recommendations for future research. J Am Coll Nutr.

[17] Murray B (2007). Hydration and physical performance. J Am Coll Nutr.

[18] Alfredo Martínez Hernández J, Cámara Hurtado M, Maria Giner Pons R, González Fandos E, López García E, AESAN Scienitific Committee (2020). Informe del Comité Científico de la Agencia Española de Seguridad Alimentaria y Nutrición (AESAN) de revisión y actualización de las Recomen-daciones Dietéticas Para la población española. Revista del Comité Científico de la AESAN.

[19] U.S. Department of Agriculture, U.S. Department of Health and Human Services (2020). Dietary guidelines for Americans, 2020-2025.

[20] EFSA Panel on Dietetic Products Nutrition and Allergies (NDA) (2010). Scientific opinion on dietary reference values for water. EFSA J.

[21] Institute of Medicine (2005). Dietary reference intakes for water, potassium, sodium, chloride, and sulfate.

[22] Gandy J (2015). Water intake: validity of population assessment and recommendations. Eur J Nutr.

[23] Risk reduction of cognitive decline and dementia: WHO guidelines. Geneva: World Health Organization; 2019. Licence: CC BY-NC-SA 3.0 IGO. 31219687

[24] Volkert D, Chourdakis M, Faxen-Irving G, Frühwald T, Landi F, Suominen MH (2015). ESPEN guidelines on nutrition in dementia. Clin Nutr.

[25] National Institute for Health and Care Excellence (NICE) (2015). Dementia, disability and frailty in later life-mid-life approaches to delay or prevent onset. NICE guideline.

[26] Armstrong-Esther CA, Browne KD, Armstrong-Esther DC, Sander L (1996). The institutionalized elderly: dry to the bone!. Int J Nurs Stud.

[27] Marra MV, Simmons SF, Shotwell MS, Hudson A, Hollingsworth EK, Long E (2016). Elevated serum osmolality and total water deficit indicate impaired hydration status in residents of long-term care facilities regardless of low or high body mass index. J Acad Nutr Diet.

[28] Hooper L, Bunn DK, Downing A, Jimoh FO, Groves J, Free C (2016). Which frail older people are dehydrated? The UK DRIE study. J Gerontol Ser A Biol Sci Med Sci.

[29] Lauriola M, Mangiacotti A, D’Onofrio G, Cascavilla L, Paris F, Paroni G (2018). Neurocognitive disorders and dehydration in older patients: clinical experience supports the hydromolecular hypothesis of dementia. Nutrients..

[30] Majdi M, Hosseini F, Naghshi S, Djafarian K, Shab-Bidar S (2021). Total and drinking water intake and risk of all-cause and cardiovascular mortality: a systematic review and dose-response meta-analysis of prospective cohort studies. Int J Clin Pract.

[31] Bialecka-Dębek A, Pietruszka B (2019). The association between hydration status and cognitive function among free-living elderly volunteers. Aging Clin Exp Res.

[32] Suhr JA, Patterson SM, Austin AW, Heffner KL (2010). The relation of hydration status to declarative memory and working memory in older adults. J Nutr Health Aging.

[33] Suhr JA, Hall J, Patterson SM, Niinistö RT (2004). The relation of hydration status to cognitive performance in healthy older adults. Int J Psychophysiol.

[34] Bethancourt HJ, Kenney WL, Almeida DM, Rosinger AY (2020). Cognitive performance in relation to hydration status and water intake among older adults, NHANES 2011–2014. Eur J Nutr.

[35] Martínez-González MA, Buil-Cosiales P, Corella D, Bulló M, Fitó M, Vioque J (2019). Cohort profile: design and methods of the PREDIMED-plus randomized trial. Int J Epidemiol.

[36] Salas-Salvadó J, Díaz-López A, Ruiz-Canela M, Basora J, Fitó M, Corella D (2019). Effect of a lifestyle intervention program with energy-restricted Mediterranean diet and exercise on weight loss and cardiovascular risk factors: one-year results of the PREDIMED-plus trial. Diabetes Care.

[37] Willett W (2013). Nutritional epidemiology, 3rd edn. Chapter 11.

[38] Fernández-Ballart JD, Piñol JL, Zazpe I, Corella D, Carrasco P, Toledo E (2010). Relative validity of a semi-quantitative food-frequency questionnaire in an elderly Mediterranean population of Spain. Br J Nutr.

[39] Palma I, Farra A, Cantós D (2008). Tablas de composición de alimentos por medidas caseras de consumo habitual en España.

[40] RedBEDCA, AESAN (2006). Base de Datos Española de Composición de Alimentos.

[41] Aranceta Bartrina J, Arija Val V, Maíz Aldalur E, Martínez de la Victoria Muñoz E, Ortega Anta RM, Grupo Colaborativo de la Sociedad Española de Nutrición Comunitaria (SENC) (2016). Dietary guidelines for the Spanish population (SENC, December 2016); the new graphic icon of healthy nutrition. Nutr Hosp.

[42] Moreiras O, Carbajal A, Cabrera L, Cuadrado C. Tablas de composición de alimentos Guía de prácticas [Food Composition Tables. Practice Guide]. 19th ed. Madrid: Piramide; 2018. p. 496.

[43] Mataix Verdú J, García Diz L, Mañas Almendros M, Martinez de Vitoria E, Llopis González J. Tablas de composición de alimentos [Food composition tables]. 5th ed. Granada; Universidad de Granada; 2013.

[44] Hooper L, Abdelhamid A, Attreed NJ, Campbell WW, Channell AM, Chassagne P, et al. Clinical symptoms, signs and tests for identification of impending and current water-loss dehydration in older people. Cochrane Database Syst Rev. 2015. 10.1002/14651858.CD009647.pub2. 10.1002/14651858.CD009647.pub2 PMC7097739 25924806

[45] Khajuria A, Krahn J (2005). Osmolality revisited - deriving and validating the best formula for calculated osmolality. Clin Biochem.

[46] Hooper L, Bunn DK, Abdelhamid A, Gillings R, Jennings A, Maas K (2016). Water-loss (intracellular) dehydration assessed using urinary tests: how well do they work? Diagnostic accuracy in older people. Am J Clin Nutr.

[47] Blesa R, Pujol M, Aguilar M, Santacruz P, Bertran-Serra I, Hernández G (2001). Clinical validity of the “mini-mental state” for Spanish speaking communities. Neuropsychologia..

[48] Folstein MF, Folstein SE, McHugh PR (1975). “Mini-mental state”. A practical method for grading the cognitive state of patients for the clinician. J Psychiatr Res.

[49] Benton AL, Hamsher K, Sivan AB (1994). Multilingual aphasia examination.

[50] Wechsler D (1997). Wechsler adult intelligence scale-III.

[51] Rossi L, Neer C-R, Lopetegui S (2008). Escala de inteligencia Para adultos de WECHSLER. WAIS-III Índice de comprensión verbal. Normas Para los subtests: Vocabulario, analogías e información, Para la ciudad de La Plata Edades: 16 a 24 Años.

[52] Aprahamian I, Martinelli JE, Neri AL, Yassuda MS (2009). O Teste do Desenho do Relógio: Revisão da acurácia no rastreamento de demência. Dementia e Neuropsychologia.

[53] Paganini-Hill A, Clark LJ (2011). Longitudinal assessment of cognitive function by clock drawing in older adults. Dement Geriatr Cogn Dis Extra.

[54] Del Ser QT, García De Yébenes MJ, Sánchez Sánchez F, Frades Payo B, Rodríguez Laso Á, Bartolomé Martínez MP (2004). Evaluación cognitiva del anciano. Datos normativos de una muestra poblacional española de más de 70 años. Med Clin (Barc).

[55] Llinàs-Reglà J, Vilalta-Franch J, López-Pousa S, Calvó-Perxas L, Torrents Rodas D, Garre-Olmo J (2017). The trail making test: association with other neuropsychological measures and normative values for adults aged 55 years and older from a Spanish-speaking population-based sample. Assessment..

[56] Brouwer-Brolsma EM, Dhonukshe-Rutten RAM, van Wijngaarden JP, van de Zwaluw NL, In’t Veld PH, Wins S (2015). Cognitive performance: a cross-sectional study on serum vitamin D and its interplay with glucose homeostasis in Dutch older adults. J Am Med Dir Assoc.

[57] Paz-Graniel I, Babio N, Becerra-Tomás N, Toledo E, Camacho-Barcia L, Corella D (2021). Association between coffee consumption and total dietary caffeine intake with cognitive functioning: cross-sectional assessment in an elderly Mediterranean population. Eur J Nutr.

[58] Molina L, Sarmiento M, Peñafiel J, Donaire D, Garcia-Aymerich J, Gomez M (2017). Validation of the REGICOR short physical activity questionnaire for the adult population. PLoS One.

[59] Paz-Graniel I, Babio N, Serra-Majem L, Vioque J, Zomeño MD, Corella D (2020). Fluid and total water intake in a senior Mediterranean population at high cardiovascular risk: demographic and lifestyle determinants in the PREDIMED-plus study. Eur J Nutr.

[60] Drewnowski A, Rehm CD, Constant F (2013). Water and beverage consumption among adults in the United States: cross-sectional study using data from NHANES 2005–2010. BMC Public Health.

[61] Polhuis KCMM, Wijnen AHC, Sierksma A, Calame W, Tieland M (2017). The diuretic action of weak and strong alcoholic beverages in elderly men: a randomized diet-controlled crossover trial. Nutrients..

[62] Xu W, Wang H, Wan Y, Tan C, Li J, Tan L (2017). Alcohol consumption and dementia risk: a dose–response meta-analysis of prospective studies. Eur J Epidemiol.

[63] Zhang Y, Coca A, Casa DJ, Antonio J, Green JM, Bishop PA (2015). Caffeine and diuresis during rest and exercise: a meta-analysis. J Sci Med Sport.

[64] van den Berg B, de Jong M, Woldorff MG, Lorist MM (2020). Caffeine boosts preparatory attention for reward-related stimulus information. J Cogn Neurosci.

[65] Chen JQA, Scheltens P, Groot C, Ossenkoppele R (2020). Associations between caffeine consumption, cognitive decline, and dementia: a systematic review. J Alzheimers Dis.

[66] Xu W, Tan CC, Zou JJ, Cao XP, Tan L (2020). Sleep problems and risk of all-cause cognitive decline or dementia: an updated systematic review and meta-analysis. J Neurol Neurosurg Psychiatry.

[67] Rosinger AY, Lawman HG, Akinbami LJ, Ogden CL (2016). The role of obesity in the relation between total water intake and urine osmolality in US adults, 2009-2012. Am J Clin Nutr.

[68] You Y, Liu Z, Chen Y, Xu Y, Qin J, Guo S (2021). The prevalence of mild cognitive impairment in type 2 diabetes mellitus patients: a systematic review and meta-analysis. Acta Diabetol.

[69] Iadecola C, Gottesman RF (2019). Neurovascular and cognitive dysfunction in hypertension: epidemiology, pathobiology, and treatment. Circ Res.

[70] An Y, Zhang X, Wang Y, Wang Y, Liu W, Wang T (2019). Longitudinal and nonlinear relations of dietary and serum cholesterol in midlife with cognitive decline: results from EMCOA study. Mol Neurodegener.

[71] John A, Patel U, Rusted J, Richards M, Gaysina D (2019). Affective problems and decline in cognitive state in older adults: a systematic review and meta-analysis. Psychol Med.

[72] Sanz J, Luis A, Carmelo P, Resumen V (2003). Adaptación española del Inventario Para la Depresión de Beck-II (BDI-II): 2. Propiedades psicométricas en población general the Spanish adaptation of Beck’s depression inventory-II (BDI-II): 2. Psychometric properties in the general population. Clínica y Salud.

[73] National Institute of Diabetes and Digestive and Kidney Diseases. Estimating glomerular filtration rate. https://www.niddk.nih.gov/health-information/professionals/clinical-tools-patient-management/kidney-disease/laboratory-evaluation/glomerular-filtration-rate/estimating. Accessed 1 Jun 2022.

[74] Dementia Care Central, National Institute on Aging. Mini-Mental State Exam (MMSE) Alzheimer’s/Dementia Test: administration, accuracy and scoring. 2020. https://www.dementiacarecentral.com/mini-mental-state-exam/. Accessed 5 Jul 2021.

[75] Masento NA, Golightly M, Field DT, Butler LT, van Reekum CM (2014). Effects of hydration status on cognitive performance and mood. Br J Nutr.

[76] Wittbrodt MT, Millard-Stafford M (2018). Dehydration impairs cognitive performance: a meta-analysis. Med Sci Sports Exerc.

[77] Kanbay M, Yilmaz S, Dincer N, Ortiz A, Sag AA, Covic A (2019). Antidiuretic hormone and serum osmolarity physiology and related outcomes: what is old, what is new, and what is unknown?. J Clin Endocrinol Metab.

[78] Insel TR (2016). Translating oxytocin neuroscience to the clinic: a National Institute of Mental Health perspective. Biol Psychiatry.

[79] Lu Q, Lai J, Du Y, Huang T, Prukpitikul P, Xu Y (2019). Sexual dimorphism of oxytocin and vasopressin in social cognition and behavior. Psychol Res Behav Manag.

[80] Bluthe R, Gheusi G, Dantzer R (1993). Gonadal steroids influence the involvement of arginine vasopressin in social recognition in mice. Psychoneuroendocrinology..

[81] Kempton MJ, Ettinger U, Schmechtlg A, Winter EM, Smith L, McMorris T (2009). Effects of acute dehydration on brain morphology in healthy humans. Hum Brain Mapp.

[82] Duning T, Kloska S, Steinstrater O, Kugel H, Heindel W, Knecht S (2005). Dehydration confounds the assessment of brain atrophy. Neurology..

[83] Trangmar S, Chiesa S, Kalsi K, Secher N, González-Alonso J (2015). Hydration and the human brain circulation and metabolism. Nutr Hosp.

[84] Rasmussen P, Nybo L, Volianitis S, Møller K, Secher NH, Gjedde A (2010). Cerebral oxygenation is reduced during hyperthermic exercise in humans. Acta Physiol.

[85] Piil JF, Lundbye-Jensen J, Trangmar SJ, Nybo L (2017). Performance in complex motor tasks deteriorates in hyperthermic humans. Temperature..

[86] Ogoh S (2017). Relationship between cognitive function and regulation of cerebral blood flow. J Physiol Sci.

[87] Ogoh S, Tsukamoto H, Hirasawa A, Hasegawa H, Hirose N, Hashimoto T. The effect of changes in cerebral blood flow on cognitive function during exercise. Physiol Rep. 2014;2(9):e12163. 10.14814/phy2.12163 PMC4270220 25263210

[88] Claassen JAHR, Thijssen DHJ, Panerai RB, Faraci FM (2021). Regulation of cerebral blood flow in humans: physiology and clinical implications of autoregulation. Physiol Rev.

[89] Hooper L, Abdelhamid A, Ali A, Bunn DK, Jennings A, John WG (2015). Diagnostic accuracy of calculated serum osmolarity to predict dehydration in older people: adding value to pathology laboratory reports. BMJ Open.

[90] Lacey J, Corbett J, Forni L, Hooper L, Hughes F, Minto G (2019). A multidisciplinary consensus on dehydration: definitions, diagnostic methods and clinical implications. Ann Med.

[91] Siervo M, Bunn D, Prado CM, Hooper L (2014). Accuracy of prediction equations for serum osmolarity in frail older people with and without diabetes. Am J Clin Nutr.

[92] Chang T, Ravi N, Plegue MA, Sonneville KR, Davis MM (2016). Inadequate hydration, BMI, and obesity among US adults: NHANES 2009-2012. Ann Fam Med.

