# Supplementary material for: Water intake, hydration status and 2-year changes in cognitive performance: a prospective cohort study
Source: BMC Med. 2023 Mar 8;21:82. doi: 10.1186/s12916-023-02771-4 (PMC9993798; doi:10.1186/s12916-023-02771-4)
Supplement: Supplementary file 1 — Additional file 1: Table S1. Hydration and water intake definitions. Table S2. Associations between cognitive assessments and water and fluid intake exposures. Table S3. Associations between cognitive assessments and EFSA fluid intake related guidelines. Table S4. Associations between cognitive assessments and hydration status. Table S5. Sensitivity analysis in global cognitive function according to water and fluid intake related exposures Table S6. Sensitivity analysis in global cognitive function according to EFSA fluid intake related guidelines. Table S7. Sensitivity analysis in global cognitive function according to hydration status. Fig. S1. Flow diagram of participants. Fig. S2. Continuous sensitivity analysis by sex. Fig. S3. Categorical sensitivity analysis by sex. [file 12916_2023_2771_MOESM1_ESM.pdf]

Hydration status, water intake and changes in cognitive performance in older adults:

A prospective cohort

ADDITIONAL FILE 1

Table of Contents

TABLES 2

Supplemental Table 1. Description of the hydration and water intake exposures assessed. 2

Supplemental Table 2. Multivariable adjusted mean 2-year change (β-coefficients and 95% CIs) in cognitive performance according to water and fluid intake related exposures in the PREDIMED-Plus cohort. 3

Supplemental Table 3. Multivariable adjusted mean 2-year change (β-coefficients and 95% CIs) in cognitive performance according to EFSA fluid intake related guidelines in the PREDIMED-Plus cohort. 11

Supplemental Table 4. Multivariable adjusted mean 2-year change (β-coefficients and 95% CIs) in cognitive performance according to hydration status in the PREDIMED-Plus cohort. 14

Supplemental Table 5. Sensitivity analysis of the multivariable adjusted mean 2-year change (β-coefficients and 95% CIs) in global cognitive function (GCF[a], n=1395) according to water and fluid intake related exposures in the PREDIMED-Plus cohort. 17

Supplemental Table 6. Sensitivity analysis of the multivariable adjusted mean 2-year change (β-coefficients and 95% CIs) in global cognitive function (GCF[a], n=1395) according to EFSA fluid intake related guidelines in the PREDIMED-Plus cohort. 19

Supplemental Table 7. Sensitivity analysis of the multivariable adjusted mean 2-year change (β-coefficients and 95% CIs) in global cognitive function (GCF[a]) according to hydration status in the PREDIMED-Plus cohort. 20

FIGURES 22

Figure 1. Flow diagram of participants in the PREDIMED-Plus sub-study for the analysis of a priori water intake and hydration status and cognitive performance in the PREDIMED-Plus trial. 22

Figure 2. Beta-coefficients and 95% CI for hydration status and water and fluid intakes continuously with 2-year changes in global cognitive function (z-scores) in women (A) and men (B). 23

Figure 3. Beta-coefficients and 95% CI for hydration status and water and fluid intakes categorically with 2-year changes in global cognitive function (z-scores) in women (A) and men (B). 24

TABLES

Supplemental Table 1. Description of the hydration and water intake exposures assessed.

Exposure	Definition
Dehydration Status	Estimated based on calculated SOSM using fasting serum glucose, blood urea nitrogen, sodium, and potassium levels according to the equation:<br><br>SOSM=1.86*(sodium + potassium) + 1.15*glucose + blood urea nitrogen + 14<br><br>*Dehydrated* was considered SOSM>300 mmol/L<br>*Impending dehydration* was considered SOSM 295 to 300 mmol/L<br>*Hydrated* was considered <295 mmol/L
Water Intake	Tap and bottled water use based on responses to the 32-item BIAQ.
Water, All Fluids	Tap and bottled water, plus water from other beverages and liquid food sources (e.g., soups) based on responses to the 32-item BIAQ.
Total Water	Water from foods based on responses to the 143-item FFQ (e.g., fruits, vegetables) plus drinking water and other beverage-related sources based on responses to the 32-item BIAQ.
EFSA TFWI	Meets recommendations for total water intake from fluids based on data from responses to the 32-item BIAQ, according to EFSA regulations and guidance[1].<br>Does Not Meet EFSA TFWI = Men: <2000 ml/d; Women <1600 ml/d)<br>AND<br>Does Meet EFSA TFWI = Men ≥2000 ml/d; Women ≥1600 ml/d
EFSA TWI	Meets recommendations for total water intake (fluid + food sources), according to EFSA regulations and guidance[1]. Water intake amounts were based on responses to the 32-item BIAQ and 143-item FFQ.<br>Does Not Meet EFSA TWI = Men: <2500 ml/d; Women <2000 ml/d)<br>AND<br>Does Meet EFSA TWI = Men ≥2500 ml/d; Women ≥2000 ml/d

Abbreviations: BIAQ, Beverage Intake Assessment Questionnaire; EFSA, European Food Safety Authority; FFQ, Food Frequency Questionnaire; d, day; SOSM, serum osmolarity; TFWI, total fluid water intake; TWI, total water intake.

[1]EFSA Panel on Dietetic Products Nutrition and Allergies (NDA). Scientific Opinion on Dietary Reference Values for water. EFSA Journal. 2010;8(3):1459–507.

Supplemental Table 2. Multivariable adjusted mean 2-year change (β-coefficients and 95% CIs) in cognitive performance according to water and fluid intake related exposures in the PREDIMED-Plus cohort.

WATER INTAKE (BEVERAGE)[a]													
		Categorical								Continuous[b]		
Intake Range (ml/day)		<500	500 to 1000		1000 to 1500		>1500		p for trend			
	N	β (95% CI)	β	(95% CI)	β	(95% CI)	β	(95% CI)		β	(95% CI)	p-value
**GCF**[c]	1395											
crude model		Reference	-0.07	(-0.22, 0.07)	-0.14	(-0.30, 0.01)	0.04	(-0.13, 0.21)	0.883	-0.00001	(-0.00011, 0.00010)	0.898
model 1		Reference	-0.01	(-0.09, 0.07)	-0.01	(-0.10, 0.08)	-0.02	(-0.12, 0.08)	0.750	-0.00001	(-0.00007, 0.00005)	0.850
model 2		Reference	0.01	(-0.08, 0.09)	-0.01	(-0.09, 0.08)	-0.02	(-0.11, 0.08)	0.669	-0.00001	(-0.00007, 0.00005)	0.716
model 3		Reference	0.01	(-0.07, 0.09)	0.00	(-0.09, 0.08)	-0.01	(-0.11, 0.09)	0.761	-0.00001	(-0.00007, 0.00005)	0.805
**MMSE**	1651											
crude model		Reference	-0.02	(-0.15, 0.10)	-0.02	(-0.15, 0.11)	0.13	(-0.02, 0.28)	0.154	0.00006	(-0.00004, 0.00016)	0.229
model 1		Reference	-0.01	(-0.12, 0.10)	0.00	(-0.11, 0.11)	0.07	(-0.06, 0.20)	0.333	0.00003	(-0.00005, 0.00012)	0.469
model 2		Reference	-0.01	(-0.12, 0.09)	0.01	(-0.10, 0.12)	0.08	(-0.06, 0.21)	0.260	0.00004	(-0.00005, 0.00012)	0.393
model 3		Reference	-0.01	(-0.12, 0.10)	0.00	(-0.11, 0.11)	0.08	(-0.06, 0.21)	0.271	0.00004	(-0.00005, 0.00012)	0.415
**CDT**	1652											
crude model		Reference	-0.07	(-0.19, 0.06)	-0.02	(-0.15, 0.10)	-0.07	(-0.23, 0.08)	0.546	-0.00002	(-0.00012, 0.00007)	0.637
model 1		Reference	-0.08	(-0.19, 0.04)	-0.04	(-0.16, 0.07)	-0.11	(-0.25, 0.04)	0.257	-0.00005	(-0.00013, 0.00004)	0.304
model 2		Reference	-0.06	(-0.18, 0.05)	-0.04	(-0.15, 0.07)	-0.11	(-0.26, 0.03)	0.195	-0.00005	(-0.00014, 0.00003)	0.219
model 3		Reference	-0.06	(-0.17, 0.06)	-0.03	(-0.14, 0.09)	-0.10	(-0.25, 0.04)	0.253	-0.00005	(-0.00014, 0.00004)	0.277
**VFT-a**	1686											
crude model		Reference	-0.15	(-0.29, -0.01)*	-0.16	(-0.31, -0.02)*	0.08	(-0.10, 0.25)	0.649	0.00001	(-0.00010, 0.00012)	0.821
model 1		Reference	-0.06	(-0.17, 0.05)	-0.08	(-0.20, 0.03)	0.07	(-0.07, 0.20)	0.592	0.00002	(-0.00007, 0.00010)	0.717
model 2		Reference	-0.05	(-0.16, 0.06)	-0.08	(-0.19, 0.04)	0.08	(-0.06, 0.21)	0.503	0.00002	(-0.00006, 0.00010)	0.671
model 3		Reference	-0.05	(-0.16, 0.06)	-0.08	(-0.20, 0.04)	0.08	(-0.06, 0.21)	0.527	0.00002	(-0.00007, 0.00010)	0.718

**VFT-p**	1686											
crude model		Reference	-0.07	(-0.21, 0.08)	-0.07	(-0.23, 0.09)	-0.01	(-0.18, 0.16)	0.863	0.00000	(-0.00011, 0.00010)	0.963
model 1		Reference	-0.02	(-0.12, 0.09)	0.00	(-0.11, 0.12)	-0.03	(-0.16, 0.10)	0.838	0.00000	(-0.00008, 0.00008)	0.993
model 2		Reference	0.00	(-0.11, 0.10)	0.01	(-0.10, 0.13)	-0.01	(-0.14, 0.11)	0.971	0.00000	(-0.00008, 0.00008)	0.930
model 3		Reference	-0.01	(-0.12, 0.09)	0.01	(-0.11, 0.12)	-0.01	(-0.14, 0.11)	0.976	0.00000	(-0.00008, 0.00008)	0.923
**TMT-A**	1683											
crude model		Reference	0.04	(-0.09, 0.16)	-0.01	(-0.14, 0.12)	-0.10	(-0.24, 0.05)	0.157	-0.00008	(-0.00017, 0.00002)	0.113
model 1		Reference	-0.01	(-0.11, 0.09)	-0.03	(-0.13, 0.08)	0.00	(-0.11, 0.11)	0.873	-0.00001	(-0.00009, 0.00006)	0.707
model 2		Reference	-0.02	(-0.12, 0.08)	-0.03	(-0.14, 0.07)	-0.01	(-0.12, 0.11)	0.784	-0.00002	(-0.00009, 0.00006)	0.672
model 3		Reference	-0.02	(-0.12, 0.08)	-0.04	(-0.14, 0.07)	-0.01	(-0.12, 0.10)	0.743	-0.00002	(-0.00009, 0.00006)	0.656
**TMT-B**	1681											
crude model		Reference	0.10	(-0.02, 0.23)	0.14	(0.003, 0.27)*	-0.07	(-0.22, 0.08)	0.738	-0.00002	(-0.00012, 0.00007)	0.668
model 1		Reference	0.08	(-0.03, 0.18)	0.06	(-0.05, 0.17)	-0.04	(-0.17, 0.08)	0.555	-0.00002	(-0.00010, 0.00006)	0.568
model 2		Reference	0.07	(-0.03, 0.17)	0.06	(-0.05, 0.17)	-0.04	(-0.16, 0.09)	0.631	-0.00001	(-0.00009, 0.00006)	0.705
model 3		Reference	0.07	(-0.03, 0.17)	0.06	(-0.05, 0.17)	-0.04	(-0.17, 0.09)	0.625	-0.00001	(-0.00009, 0.00006)	0.720
**DST-f**	1449											
crude model		Reference	0.15	(-0.0005, 0.30)	0.13	(-0.02, 0.29)	0.30	(0.11, 0.49)*	**0.004**	0.00017	(-0.00006, 0.00029)	**0.002**
model 1		Reference	0.06	(-0.06, 0.17)	0.09	(-0.04, 0.21)	0.15	(-0.002, 0.31)	**0.047**	0.00010	(-0.00001, 0.00019)	**0.031**
model 2		Reference	0.08	(-0.04, 0.20)	0.11	(-0.02, 0.24)	0.16	(0.01, 0.31)*	**0.029**	0.00011	(-0.00002, 0.00020)	**0.021**
model 3		Reference	0.09	(-0.03, 0.21)	0.11	(-0.01, 0.24)	0.17	(0.02, 0.32)*	**0.021**	0.00011	(-0.00002, 0.00020)	**0.016**
**DST-b**	1449											
crude model		Reference	0.15	(0.003, 0.30)*	0.07	(-0.08, 0.23)	0.19	(0.01, 0.38)*	0.132	0.00010	(-0.00001, 0.00021)	0.079
model 1		Reference	0.06	(-0.06, 0.18)	0.02	(-0.11, 0.14)	0.02	(-0.13, 0.17)	0.970	0.00002	(-0.00007, 0.00011)	0.635
model 2		Reference	0.08	(-0.04, 0.20)	0.04	(-0.08, 0.16)	0.05	(-0.10, 0.19)	0.740	0.00003	(-0.00006, 0.00012)	0.481
model 3		Reference	0.08	(-0.04, 0.20)	0.04	(-0.08, 0.16)	0.05	(-0.10. 0.20)	0.691	0.00004	(-0.00005, 0.00012)	0.434

		**WATER FROM ALL FLUIDS[d]**										
		Quantiles of Water from All Fluids								Continuous		
		Q1	Q2		Q3		Q4		p for trend			
	N	β (95% CI)	β	(95% CI)	β	(95% CI)	β	(95% CI)		β	(95% CI)	p-value
**GCF**	1395	1127[e] (251 to 1441)	1623 (1445 to 1808)		2005 (1809 to 2222)		2608 (2225 to 3722)					
crude model		Reference	0.02	(-0.13, 0.17)	0.00	(-0.15, 0.15)	0.13	(-0.01, 0.28)	0.085	0.00007	(-0.00002, 0.00015)	0.122
model 1		Reference	-0.03	(-0.11, 0.06)	-0.04	(-0.13, 0.05)	0.00	(-0.08, 0.08)	0.980	-0.00001	(-0.00006, 0.00004)	0.648
model 2		Reference	-0.03	(-0.11, 0.06)	-0.05	(-0.14, 0.04)	-0.01	(-0.09, 0.08)	0.851	-0.00002	(-0.00007, 0.00003)	0.499
model 3		Reference	-0.02	(-0.11, 0.06)	-0.05	(-0.13, 0.04)	0.00	(-0.09, 0.08)	0.870	-0.00002	(-0.00007, 0.00003)	0.506
**MMSE**	1651	1121 (251 to 1443)	1621 (1445 to 1808)		2005 (1809 to 2222)		2606 (2225 to 3951)					
crude model		Reference	0.00	(-0.13, 0.13)	-0.02	(-0.15, 0.12)	0.13	(0.003, 0.25)*	**0.049**	0.00007	(-0.000003, 0.00015)	0.059
model 1		Reference	-0.06	(-0.18, 0.05)	-0.07	(-0.19, 0.04)	0.03	(-0.08, 0.14)	0.555	0.00001	(-0.00006, 0.00007)	0.844
model 2		Reference	-0.06	(-0.17, 0.06)	-0.07	(-0.18, 0.04)	0.03	(-0.08, 0.14)	0.599	0.00001	(-0.00006, 0.00007)	0.875
model 3		Reference	-0.06	(-0.17, 0.06)	-0.07	(-0.18, 0.05)	0.03	(-0.08, 0.14)	0.577	0.00001	(-0.00006, 0.00007)	0.856
**CDT**	1652	1119 (251 to 1443)	1620 (1445 to 1808)		2005 (1809 to 2222)		2605 (2225 to 3951)					
crude model		Reference	0.10	(-0.02, 0.23)	0.01	(-0.12, 0.14)	0.11	(-0.02, 0.23)	0.220	0.00007	(-0.000002, 0.00014)	0.055
model 1		Reference	0.08	(-0.03, 0.19)	-0.02	(-0.15, 0.10)	0.03	(-0.09, 0.14)	0.984	0.00002	(-0.00005, 0.00009)	0.535
model 2		Reference	0.07	(-0.04, 0.19)	-0.03	(-0.16, 0.09)	0.02	(-0.10, 0.13)	0.840	0.00001	(-0.00005, 0.00008)	0.687
model 3		Reference	0.08	(-0.04, 0.19)	-0.03	(-0.16, 0.09)	0.00	(-0.12, 0.11)	0.575	0.00000	(-0.00007, 0.00007)	0.984

**VFT-a**	1686	1118 (251 to 1443)	1620 (1445 to 1808)		2005 (1809 to 2222)		2606 (2225 to 3951)					
crude model		Reference	-0.02	(-0.17, 0.12)	0.01	(-0.13, 0.15)	0.11	(-0.04, 0.25)	0.113	0.00005	(-0.00007, 0.00007)	0.241
model 1		Reference	-0.06	(-0.17, 0.05)	-0.04	(-0.15, 0.07)	-0.03	(-0.15, 0.08)	0.642	-0.00003	(-0.00009, 0.00004)	0.383
model 2		Reference	-0.06	(-0.17, 0.05)	-0.04	(-0.15, 0.07)	-0.04	(-0.15, 0.07)	0.558	-0.00003	(-0.00010, 0.00003)	0.323
model 3		Reference	-0.06	(-0.17, 0.05)	-0.04	(-0.15, 0.07)	-0.04	(-0.15, 0.07)	0.541	-0.00004	(-0.00010, 0.00003)	0.305
**VFT-p**	1686	1118 (251 to 1443)	1620 (1445 to 1808)		2005 (1809 to 2222)		2606 (2225 to 3951)					
crude model		Reference	0.09	(-0.06, 0.23)	0.06	(-0.08, 0.21)	0.19	(0.05, 0.34)*	**0.013**	0.00010	(-0.00001, 0.00019)	**0.030**
model 1		Reference	0.08	(-0.02, 0.18)	0.01	(-0.09, 0.12)	0.07	(-0.03, 0.18)	0.338	0.00002	(-0.00004, 0.00009)	0.511
model 2		Reference	0.08	(-0.02, 0.17)	0.01	(-0.10, 0.12)	0.07	(-0.04, 0.17)	0.367	0.00002	(-0.00005, 0.00009)	0.558
model 3		Reference	0.08	(-0.02, 0.18)	0.02	(-0.09, 0.13)	0.09	(-0.02, 0.20)	0.196	0.00003	(-0.00003, 0.00010)	0.328
**TMT-A**	1683	1118 (251 to 1443)	1620 (1445 to 1808)		2005 (1809 to 2222)		2605 (2225 to 3951)					
crude model		Reference	-0.11	(-0.24, 0.02)	-0.09	(-0.23, 0.04)	-0.21	(-0.34, -0.07)*	**0.003**	-0.00010	(-0.00018, 0.00003)	**0.009**
model 1		Reference	-0.03	(-0.13, 0.07)	-0.03	(-0.14, 0.08)	-0.02	(-0.12, 0.07)	0.664	0.00000	(-0.00006, 0.00006)	0.929
model 2		Reference	-0.03	(-0.14, 0.07)	-0.02	(-0.13, 0.08)	-0.02	(-0.12, 0.08)	0.750	0.00001	(-0.00005, 0.00007)	0.821
model 3		Reference	-0.03	(-0.14, 0.07)	-0.02	(-0.12, 0.09)	-0.02	(-0.12, 0.08)	0.847	0.00001	(-0.00005, 0.00007)	0.724
**TMT-B**	1681	1118 (251 to 1443)	1620 (1445 to 1808)		2005 (1809 to 2222)		2606 (2225 to 3951)					
crude model		Reference	0.08	(-0.05, 0.21)	0.00	(-0.13, 0.13)	-0.06	(-0.19, 0.06)	0.181	-0.00004	(-0.00012, 0.00004)	0.335
model 1		Reference	0.07	(-0.03, 0.17)	0.02	(-0.09, 0.13)	0.00	(-0.11, 0.10)	0.731	0.00000	(-0.00006, 0.00007)	0.924
model 2		Reference	0.08	(-0.02, 0.18)	0.04	(-0.06, 0.15)	0.02	(-0.09, 0.12)	0.976	0.00002	(-0.00005, 0.00008)	0.610
model 3		Reference	0.08	(-0.02, 0.18)	0.05	(-0.06, 0.15)	0.03	(-0.08, 0.13)	0.802	0.00002	(-0.00004, 0.00009)	0.469

**DST-f**	1449	1122 (251 to 1442)	1624 (1445 to 1808)		2005 (1809 to 2222)		2609 (2225 to 3722)					
crude model		Reference	0.11	(-0.04, 0.25)	0.14	(-0.01, 0.30)	0.27	(0.12, 0.42)*	**0.000**	0.00017	(-0.00008, 0.00025)	**<0.001**
model 1		Reference	0.01	(-0.10, 0.13)	0.04	(-0.09, 0.16)	0.12	(0.01, 0.24)*	**0.032**	0.00007	(0.00000, 0.00014)	**0.048**
model 2		Reference	0.01	(-0.11, 0.12)	0.03	(-0.09, 0.15)	0.12	(0.01, 0.24)*	**0.030**	0.00007	(0.00000, 0.00014)	**0.047**
model 3		Reference	0.00	(-0.11, 0.12)	0.03	(-0.10, 0.15)	0.12	(0.001, 0.24)*	**0.041**	0.00007	(-0.000005, 0.00014)	0.068
**DST-b**	1449	1122 (251 to 1442)	1624 (1445 to 1808)		2005 (1809 to 2222)		2609 (2225 to 3722)					
crude model		Reference	0.09	(-0.06, 0.23)	0.10	(-0.05, 0.24)	0.17	(0.03, 0.32)*	**0.025**	0.00009	(-0.000002, 0.00017)	0.057
model 1		Reference	-0.02	(-0.13, 0.10)	-0.03	(-0.15, 0.09)	0.04	(-0.08, 0.16)	0.496	0.00001	(-0.00007, 0.00008)	0.855
model 2		Reference	-0.02	(-0.13, 0.10)	-0.03	(-0.15, 0.09)	0.04	(-0.08, 0.15)	0.505	0.00001	(-0.00006, 0.00008)	0.846
model 3		Reference	-0.01	(-0.13, 0.10)	-0.03	(-0.15, 0.09)	0.03	(-0.09, 0.15)	0.636	0.00000	(-0.00008, 0.00007)	0.955

TOTAL WATER[f]

		Quantiles of Total Water Intake								Continuous		
		Q1	Q2		Q3		Q4					
	N	β (95% CI)	β	(95% CI)	β	(95% CI)	β	(95% CI)	p for trend	β	(95% CI)	p-value
**GCF**	1395	2068 (1155 to 2395)	2616 (2400 to 2832)		3059 (2834 to <3308)		3752 (3308 to 5184)					
crude model		Reference	-0.02	(-0.17, 0.13)	-0.02	(-0.18, 0.13)	0.02	(-0.13, 0.16)	0.792	-0.00001	(-0.00008, 0.00007)	0.817
model 1		Reference	0.01	(-0.07, 0.09)	-0.06	(-0.15, 0.03)	-0.02	(-0.10, 0.07)	0.439	-0.00002	(-0.00006, 0.00002)	0.382
model 2		Reference	0.00	(-0.08, 0.09)	-0.06	(-0.15, 0.02)	-0.03	(-0.12, 0.05)	0.264	-0.00003	(-0.00007, 0.00002)	0.201
model 3		Reference	0.00	(-0.08, 0.09)	-0.07	(-0.16, 0.02)	-0.05	(-0.14, 0.04)	0.137	-0.00004	(-0.00008, 0.00001)	0.088

		2063	2615		3058		3741					
**MMSE**	1651	(1015 to 2395)	(2396 to 2832)		(2832 to <3308)		(3308 to 5184)					
crude model		Reference	0.00	(-0.13, 0.13)	0.00	(-0.14, 0.13)	0.08	(-0.05, 0.21)	0.214	0.00003	(-0.00005, 0.00010)	0.496
model 1		Reference	0.02	(-0.10, 0.13)	-0.07	(-0.19, 0.05)	0.04	(-0.07, 0.16)	0.705	0.00000	(-0.00006, 0.00006)	0.992
model 2		Reference	0.02	(-0.10, 0.13)	-0.07	(-0.19, 0.05)	0.04	(-0.07, 0.15)	0.705	0.00000	(-0.00006, 0.00006)	0.984
model 3		Reference	0.02	(-0.10, 0.13)	-0.07	(-0.19, 0.05)	0.04	(-0.07, 0.15)	0.776	-0.00001	(-0.00007, 0.00006)	0.871
		2061	2614		3058		3741					
**CDT**	1652	(1015 to 2395)	(2396 to 2832)		(>2832 to <3308)		(3308 to 5184)					
crude model		Reference	-0.01	(-0.13, 0.12)	0.05	(-0.08, 0.17)	0.04	(-0.08, 0.16)	0.380	0.00003	(-0.00004, 0.00009)	0.418
model 1		Reference	0.00	(-0.12, 0.11)	0.03	(-0.09, 0.14)	0.02	(-0.09, 0.14)	0.590	0.00001	(-0.00005, 0.00007)	0.716
model 2		Reference	-0.01	(-0.13, 0.11)	0.01	(-0.10, 0.13)	0.01	(-0.11, 0.12)	0.816	0.00000	(-0.00006, 0.00006)	0.976
model 3		Reference	-0.01	(-0.13, 0.10)	0.00	(-0.12, 0.12)	-0.03	(-0.15, 0.09)	0.712	-0.00002	(-0.00009, 0.00004)	0.520
		2063	2614		3059		3745					
**VFT-a**	1686	(1015 to 2395)	(2396 to 2832)		(>2832 to <3308)		(3308 to 5184)					
crude model		Reference	-0.06	(-0.21, 0.08)	0.06	(-0.08, 0.20)	0.03	(-0.12, 0.17)	0.415	0.00003	(-0.00005, 0.00010)	0.497
model 1		Reference	-0.05	(-0.16, 0.06)	-0.03	(-0.14, 0.08)	-0.06	(-0.17, 0.05)	0.392	-0.00003	(-0.00009, 0.00002)	0.251
model 2		Reference	-0.05	(-0.16, 0.05)	-0.03	(-0.14, 0.08)	-0.06	(-0.17, 0.05)	0.344	-0.00004	(-0.00010, 0.00002)	0.215
model 3		Reference	-0.06	(-0.17, 0.05)	-0.04	(-0.15, 0.07)	-0.08	(-0.20, 0.04)	0.236	-0.00005	(-0.00011, 0.00001)	0.125
		2063	2614		3059		3745					
**VFT-p**	1686	(1015 to 2395)	(2396 to 2832)		(>2832 to <3308)		(3308 to 5184)					
crude model		Reference	0.06	(-0.08, 0.20)	0.05	(-0.09, 0.20)	0.09	(-0.06, 0.23)	0.275	0.00005	(-0.00003, 0.00013)	0.225
model 1		Reference	0.05	(-0.03, 0.17)	0.02	(-0.09, 0.13)	-0.01	(-0.12, 0.10)	0.594	0.00000	(-0.00006, 0.00006)	0.907
model 2		Reference	0.07	(-0.03, 0.17)	0.02	(-0.09, 0.13)	-0.01	(-0.12, 0.09)	0.563	-0.00001	(-0.00007, 0.00005)	0.847
model 3		Reference	0.07	(-0.03, 0.17)	0.02	(-0.08, 0.13)	0.00	(-0.11, 0.11)	0.745	0.00000	(-0.00006, 0.00007)	0.904

**TMT-A**	1683	2062 (1015 to 2395)	2614 (2396 to 2832)		3058 (>2832 to <3308)		3744 (3308 to 5184)					
crude model		Reference	-0.06	(-0.19, 0.07)	-0.12	(-0.25, 0.01)	-0.09	(-0.22, 0.05)	0.154	-0.00004	(-0.00011, 0.00003)	0.252
model 1		Reference	-0.05	(-0.15, 0.05)	-0.05	(-0.15, 0.05)	-0.01	(-0.12, 0.10)	0.936	0.00001	(-0.00005, 0.00007)	0.781
model 2		Reference	-0.04	(-0.14, 0.06)	-0.04	(-0.14, 0.06)	0.00	(-0.11, 0.11)	0.880	0.00002	(-0.00004, 0.00007)	0.592
model 3		Reference	-0.04	(-0.14, 0.06)	-0.04	(-0.14, 0.06)	0.02	(-0.10, 0.13)	0.681	0.00003	(-0.00003, 0.00009)	0.382
**TMT-B**	1681	2063 (1015 to 2395)	2614 (2396 to 2832)		3059 (>2832 to <3308)		3745 (3308 to 5184)					
crude model		Reference	0.07	(-0.06, 0.20)	-0.03	(-0.16, 0.10)	-0.01	(-0.14, 0.12)	0.600	-0.00001	(-0.00008, 0.00006)	0.686
model 1		Reference	0.07	(-0.04, 0.17)	-0.01	(-0.12, 0.10)	0.01	(-0.10, 0.11)	0.786	0.00000	(-0.00006, 0.00006)	0.984
model 2		Reference	0.08	(-0.03, 0.18)	0.01	(-0.10, 0.11)	0.03	(-0.07, 0.13)	0.840	0.00001	(-0.00004, 0.00007)	0.667
model 3		Reference	0.08	(-0.02, 0.18)	0.02	(-0.08, 0.12)	0.06	(-0.05, 0.16)	0.498	0.00003	(-0.00003, 0.00009)	0.320
**DST-f**	1449	2068 (1155 to 2395)	2615 (2401 to 2832)		3060 (2834 to <3308)		3754 (3308 to 5184)					
crude model		Reference	0.13	(-0.03, 0.28)	0.06	(-0.10, 0.21)	0.15	(-0.01, 0.30)	0.128	0.00008	(-0.000002, 0.00016)	0.057
model 1		Reference	0.06	(-0.06, 0.18)	-0.01	(-0.13, 0.11)	0.06	(-0.07, 0.18)	0.584	0.00005	(-0.00002, 0.00011)	0.160
model 2		Reference	0.06	(-0,05, 0.18)	-0.01	(-0.13, 0.11)	0.05	(-0.07, 0.18)	0.618	0.00004	(-0.00002, 0.00011)	0.175
model 3		Reference	0.06	(-0.06, 0.18)	0.00	(-0.13, 0.12)	0.06	(-0.07, 0.19)	0.547	0.00005	(-0.00002, 0.00012)	0.144
**DST-b**	1449	2068 (1155 to 2395)	2615 (2401 to 2832)		3060 (2834 to <3308)		3754 (3308 to 5184)					
crude model		Reference	0.00	(-0,15, 0.15)	0.03	(-0.12, 0.18)	0.02	(-0.13, 0.16)	0.780	0.00000	(-0.00008, 0.00008)	0.985
model 1		Reference	-0.04	(-0.16, 0.08)	-0.07	(-0.19, 0.04)	-0.04	(-0.15, 0.08)	0.513	-0.00001	(-0.00007, 0.00006)	0.860
model 2		Reference	-0.03	(-0.15, 0.09)	-0.07	(-0.18, 0.05)	-0.03	(-0.15, 0.08)	0.495	-0.00001	(-0.00007, 0.00005)	0.789
model 3		Reference	-0.03	(-0.15, 0.09)	-0.08	(-0.19, 0.03)	-0.06	(-0.18, 0.06)	0.272	-0.00002	(-0.00009, 0.00004)	0.463

Multivariable linear regression was used to assess longitudinal association to compare the 2-year changes in cognitive function across categories of hydration status and various modes of water/fluid intake, as well as continuous associations between cognitive function variables and hydration status/fluid intake.

Multivariable model 1: adjusted for baseline corresponding cognitive function score, age (years), sex, intervention PREDIMED-Plus randomized group, and participating center (≤100, 100 to <200, 200 to <300, >300 participants).

Multivariable model 2: additionally adjusted for body mass index (kg/m2), educational level (primary, secondary or college), civil status (single, divorced or separated, married, widower), smoking habit (current, former, or never), and physical activity (METs/min/day).

Multivariable model 3: additionally adjusted for sleep status (hours per day), depressive symptomatology (yes/no), diabetes prevalence (yes/no), hypertension (yes/no), hypercholesterolemia (yes/no), energy intake (kcal/day), alcohol consumption in g/day (and adding the quadratic term), and caffeine intake (mg/day).

[a]Water Intake represents fluid water consumed as a beverage, including tap and bottled forms of water.

[b]Continuous analysis based on 100 ml increments of water intake from the noted sources.

[c]GCF represents global cognitive function, and was calculated using a composite of z-scores using the formula: GCF=(zMMSE + zCDT + zVFT-a + zVFT-b + (–zTMT-A) + (–zTMT-B) + zDST-f + zDST-b) / 8.

Where MMSE, CDT, VFT-a, VFT-p, TMT-A, TMT-B, DST-f, and DST-b are various cognitive function tests that were included in the battery of neuropsychological tests.

[d]Water from all fluids represents water consumed from all beverages.

[e]The first row of each cognitive performance assessment from this point on provides the mean and range (ml/day) of the respective fluid exposure for each quantile indicated.

[f]Total Water represents water consumed via beverage and food sources.

*Indicates within group significance (p<0.05).

Abbreviations: CDT, Clock Drawing Test; DST-b, Digit Span test-backward; DST-f, Digit Span test -forward; GCF, Global Cognitive Function; MMSE, Mini-Mental State Examination; Q, quantile; TMT-A, Trail Making Test Part A; TMT-B, Trail Making Test Part B; VFT-a, Verbal Fluency tasks semantical; VFT-p, Verbal Fluency tasks phonological.

Supplemental Table 3. Multivariable adjusted mean 2-year change (β-coefficients and 95% CIs) in cognitive performance according to EFSA fluid intake related guidelines in the PREDIMED-Plus cohort.

EFSA GUIDANCE[a] FOR TOTAL FLUID WATER INTAKE (EFSA TFWI)					
Total Fluid Water Intake<br>Mean, Range (ml/day)	N	Does Not Meet Recommendations<br>1384 (251 to 1997)<br>β (95% CI)	Meets Recommendations<br>2271 (1601 to 4398)<br>β	(95% CI)	p for trend
**GCF[b]**	1395				
crude model		Reference	-0.11	(-0.22, -0.01)	**0.037**
model 1		Reference	-0.02	(-0.08, 0.04)	0.481
model 2		Reference	-0.02	(-0.08, 0.04)	0.446
model 3		Reference	-0.03	(-0.09, 0.03)	0.375
**MMSE**	1651				
crude model		Reference	-0.01	(-0.10, 0.08)	0.781
model 1		Reference	0.03	(-0.05, 0.11)	0.439
model 2		Reference	0.03	(-0.05, 0.12)	0.407
model 3		Reference	0.03	(-0.05, 0.11)	0.467
**CDT**	1652				
crude model		Reference	0.02	(-0.06, 0.11)	0.578
model 1		Reference	0.05	(-0.03, 0.13)	0.233
model 2		Reference	0.05	(-0.04, 0.13)	0.277
model 3		Reference	0.03	(-0.05, 0.11)	0.489
**VFT-a**	1686				
crude model		Reference	-0.06	(-0.17, 0.04)	0.206
model 1		Reference	-0.02	(-0.09, 0.06)	0.684
model 2		Reference	-0.01	(-0.09, 0.06)	0.729
model 3		Reference	-0.02	(-0.10, 0.06)	0.608
**VFT-p**	1686				
crude model		Reference	0.02	(-0.09, 0.12)	0.770
model 1		Reference	0.00	(-0.08, 0.08)	0.998
model 2		Reference	0.01	(-0.07, 0.08)	0.865
model 3		Reference	0.02	(-0.06, 0.09)	0.667
**TMT-A**	1683				
crude model		Reference	-0.02	(-0.10, 0.07)	0.721
model 1		Reference	0.01	(-0.06, 0.08)	0.784
model 2		Reference	0.01	(-0.06, 0.08)	0.824
model 3		Reference	0.01	(-0.06, 0.09)	0.696
**TMT-B**	1681				
crude model		Reference	0.09	(-0.003, 0.18)	0.059
model 1		Reference	0.00	(-0.08, 0.07)	0.900
model 2		Reference	0.00	(-0.07, 0.07)	0.960
model 3		Reference	0.01	(-0.06, 0.09)	0.714

**DST-f**	1449				
crude model		Reference	-0.02	(-0.13, 0.09)	0.693
model 1		Reference	0.02	(-0.07, 0.11)	0.693
model 2		Reference	0.02	(-0.06, 0.11)	0.582
model 3		Reference	0.02	(-0.07, 0.11)	0.663
**DST-b**	1449				
crude model		Reference	-0.06	(-0.17, 0.04)	0.241
model 1		Reference	-0.03	(-0.12, 0.05)	0.431
model 2		Reference	-0.03	(-0.11, 0.06)	0.548
model 3		Reference	-0.03	(-0.12, 0.05)	0.430

**EFSA GUIDANCE[a] FOR TOTAL WATER INTAKE (EFSA TWI)**					
Total Water Intake<br>Mean, Range (ml/day)		Does Not Meet Recommendations<br>2054 (1015 to 2499)	Meets Recommendations<br>3073 (2002 to 5794)		
	N	β (95% CI)	β	(95% CI)	p for trend
**GCF[b]**	1395				
crude model		Reference	-0.25	(-0.38, -0.12)	**<0.001**
model 1		Reference	-0.05	(-0.13, 0.03)	0.233
model 2		Reference	-0.05	(-0.13, 0.02)	0.165
model 3		Reference	-0.06	(-0.14, 0.02)	0.116
**MMSE**	1651				
crude model		Reference	-0.07	(-0.19, 0.04)	0.229
model 1		Reference	0.00	(-0.10, 0.10)	0.989
model 2		Reference	0.00	(-0.10, 0.11)	0.935
model 3		Reference	0.00	(-0.10, 0.11)	0.935
**CDT**	1652				
crude model		Reference	-0.11	(-0.22, -0.01)	**0.036**
model 1		Reference	-0.03	(-0.13, 0.07)	0.555
model 2		Reference	-0.04	(-0.14, 0.06)	0.393
model 3		Reference	-0.06	(-0.17, 0.04)	0.224
**VFT-a**	1686				
crude model		Reference	-0.15	(-0.27, -0.02)	**0.025**
model 1		Reference	-0.03	(-0.13, 0.07)	0.561
model 2		Reference	-0.03	(-0.13, 0.07)	0.539
model 3		Reference	-0.04	(-0.14, 0.07)	0.468
**VFT-p**	1686				
crude model		Reference	-0.06	(-0.19, 0.07)	0.386
model 1		Reference	-0.02	(-0.12, 0.08)	0.704
model 2		Reference	-0.02	(-0.12, 0.08)	0.692
model 3		Reference	-0.01	(-0.11, 0.09)	0.787
**TMT-A**	1683				
crude model		Reference	0.04	(-0.08, 0.16)	0.526
model 1		Reference	-0.05	(-0.15, 0.05)	0.296
model 2		Reference	-0.05	(-0.14, 0.05)	0.344
model 3		Reference	-0.04	(-0.14, 0.05)	0.394

**TMT-B**	1681				
crude model		Reference	0.15	(0.04, 0.26)	**0.007**
model 1		Reference	-0.01	(-0.10, 0.09)	0.877
model 2		Reference	0.01	(-0.09, 0.10)	0.891
model 3		Reference	0.02	(-0.07, 0.11)	0.684
**DST-f**	1449				
crude model		Reference	-0.13	(-0.28, 0.01)	0.069
model 1		Reference	-0.02	(-0.13, 0.10)	0.783
model 2		Reference	-0.02	(-0.13, 0.09)	0.762
model 3		Reference	-0.01	(-0.13, 0.10)	0.833
**DST-b**	1449				
crude model		Reference	-0.18	(-0.32, -0.04)	**0.014**
model 1		Reference	-0.04	(-0.15, 0.07)	0.473
model 2		Reference	-0.04	(-0.15, 0.07)	0.504
model 3		Reference	-0.05	(-0.16, 0.06)	0.362

Multivariable linear regression was used to assess longitudinal association to compare the 2-year changes in cognitive function across categories of hydration status and various modes of water/fluid intake, as well as continuous associations between cognitive function variables and hydration status/fluid intake.

Multivariable model 1: adjusted for baseline corresponding cognitive function score, age (years), sex, intervention PREDIMED-Plus randomized group, and participating center (≤100, 100 to <200, 200 to <300, >300 participants).

Multivariable model 2: additionally adjusted for body mass index (kg/m2), educational level (primary, secondary or college), civil status (single, divorced or separated, married, widower), smoking habit (current, former, or never), and physical activity (METs/min/day).

Multivariable model 3: additionally adjusted for sleep status (hours per day), depressive symptomatology (yes/no), diabetes prevalence (yes/no), hypertension (yes/no), hypercholesterolemia (yes/no), energy intake (kcal/day), alcohol consumption in g/day (and adding the quadratic term), and caffeine intake (mg/day).

[a]Based on the regulations and guidance of the EFSA Panel on Dietetic Products, Nutrition, and Allergies (NDA): Scientific Opinion on Dietary reference values for water. Where EFSA TFWI refers to the recommended levels of total fluid water intake for older adults at 2.0 L/day and 1.6 L/day for men and women, respectively, and EFSA TWI refers to the recommended levels of total water intake, from fluids and foods, for older adults at 2.5 L/day and 2.0 L/day for men and women, respectively.

[b]GCF represents global cognitive function, and was calculated using a composite of z-scores using the formula: GCF=(zMMSE + zCDT + zVFT-a + zVFT-b + (–zTMT-A) + (–zTMT-B) + zDST-f + zDST-b) / 8.

Where MMSE, CDT, VFT-a, VFT-p, TMT-A, TMT-B, DST-f, and DST-b are various cognitive function tests that were included in the battery of neuropsychological tests.

Abbreviations: CDT, Clock Drawing Test; DST-b, Digit Span test-backward; DST-f, Digit Span test - forward; EFSA, European Food Safety Authority; GCF, Global Cognitive Function; MMSE, Mini-Mental State Examination; Q, quantile; TMT-A, Trail Making Test Part A; TMT-B, Trail Making Test Part B; VFT-a, Verbal Fluency tasks semantical; VFT-p, Verbal Fluency tasks phonological.

Supplemental Table 4. Multivariable adjusted mean 2-year change (β-coefficients and 95% CIs) in cognitive performance according to hydration status in the PREDIMED-Plus cohort.

HYDRATION STATUS (SOSM[a])										
		Categorical						Continuous		
**SOSM (mmol/L, range)**		263 to 294	295 to 299		≥300					
	N	β (95% CI)	β	(95% CI)	β	(95% CI)	p for trend	β	(95% CI)	p-value
**GCF[b]**	877									
crude model		Reference	0.11	(-0.14, 0.37)	-0.08	(-0.32, 0.17)	0.073	-0.013	(-0.026, -0.001)	**0.041**
model 1		Reference	-0.05	(-0.18, 0.09)	-0.09	(-0.22, 0.05)	0.147	-0.009	(-0.016, -0.002)	**0.008**
model 2		Reference	-0.06	(-0.19, 0.08)	-0.09	(-0.22, 0.03)	0.110	-0.009	(-0.016, -0.003)	**0.006**
model 3		Reference	-0.06	(-0.19, 0.08)	-0.11	(-0.24, 0.02)	0.058	-0.010	(-0.017, -0.004)	**0.002**
**MMSE**	996									
crude model		Reference	0.12	(-0.10, 0.33)	0.08	(-0.13, 0.29)	0.774	0.005	(-0.006, 0.015)	0.355
model 1		Reference	0.11	(-0.08, 0.30)	0.08	(-0.11, 0.26)	0.747	0.005	(-0.004, 0.014)	0.301
model 2		Reference	0.11	(-0.08, 0.29)	0.08	(-0.10, 0.26)	0.738	0.005	(-0.004, 0.014)	0.289
model 3		Reference	0.10	(-0.08, 0.29)	0.07	(-0.12, 0.26)	0.814	0.005	(-0.005, 0.015)	0.298
**CDT**	997									
crude model		Reference	0.08	(-0.14, 0.30)	0.04	(-0.17, 0.25)	0.956	-0.006	(-0.016, 0.004)	0.236
model 1		Reference	0.06	(-0.13, 0.25)	0.03	(-0.16, 0.22)	0.989	-0.007	(-0.017, 0.003)	0.159
model 2		Reference	0.05	(-0.15, 0.24)	0.02	(-0.17, 0.21)	0.997	-0.007	(-0.017, 0.003)	0.193
model 3		Reference	0.04	(-0.16, 0.24)	0.02	(-0.18, 0.21)	0.944	-0.007	(-0.017, 0.004)	0.200
**VFT-a**	1004									
crude model		Reference	-0.11	(-0.35, 0.13)	-0.14	(-0.37, 0.08)	0.230	-0.004	(-0.017, 0.009)	0.557
model 1		Reference	-0.11	(-0.30, 0.09)	-0.15	(-0.34, 0.05)	0.148	-0.009	(-0.019, 0.001)	0.067
model 2		Reference	-0.12	(-0.31, 0.07)	-0.15	(-0.34, 0.03)	0.143	-0.009	(-0.019, 0.000)	0.061
model 3		Reference	-0.12	(-0.31, 0.07)	-0.15	(-0.34, 0.05)	0.172	-0.009	(-0.019, 0.001)	0.091

**VFT-p**	1004									
crude model		Reference	-0.03	(-0.25, 0.19)	-0.19	(-0.40, 0.03)	**0.016**	-0.010	(-0.023, 0.002)	0.091
model 1		Reference	-0.06	(-0.24, 0.11)	-0.06	(-0.23, 0.11)	0.668	0.001	(-0.008, 0.010)	0.758
model 2		Reference	-0.07	(-0.24, 0.10)	-0.06	(-0.23, 0.10)	0.609	0.000	(-0.008, 0.009)	0.929
model 3		Reference	-0.08	(-0.25, 0.10)	-0.08	(-0.25, 0.09)	0.491	0.000	(-0.010, 0.009)	0.921
**TMT-A**	1002									
crude model		Reference	-0.12	(-0.37, 0.13)	0.00	(-0.25, 0.24)	0.359	0.004	(-0.007, 0.016)	0.466
model 1		Reference	-0.09	(-0.27, 0.10)	-0.06	(-0.25, 0.13)	0.806	0.000	(-0.010, 0.010)	0.991
model 2		Reference	-0.07	(-0.26, 0.11)	-0.05	(-0.24, 0.14)	0.890	0.001	(-0.009, 0.010)	0.909
model 3		Reference	-0.08	(-0.27, 0.11)	-0.06	(-0.25, 0.13)	0.784	0.000	(-0.010, 0.009)	0.937
**TMT-B**	999									
crude model		Reference	-0.15	(-0.40, 0.11)	-0.06	(-0.31, 0.19)	0.822	0.007	(-0.006, 0.019)	0.315
model 1		Reference	-0.08	(-0.30, 0.14)	-0.12	(-0.33, 0.10)	0.234	0.001	(-0.011, 0.013)	0.921
model 2		Reference	-0.08	(-0.29, 0.13)	-0.12	(-0.33, 0.09)	0.209	0.000	(-0.011, 0.012)	0.963
model 3		Reference	-0.09	(-0.30, 0.12)	-0.13	(-0.34, 0.07)	0.172	0.000	(-0.012, 0.012)	0.987
**DST-f**	901									
crude model		Reference	0.05	(-0.25, 0.35)	0.03	(-0.26, 0.32)	0.974	0.001	(-0.013, 0.014)	0.939
model 1		Reference	-0.05	(-0.25, 0.14)	-0.07	(-0.26, 0.12)	0.488	-0.004	(-0.014, 0.006)	0.435
model 2		Reference	-0.08	(-0.28, 0.11)	-0.10	(-0.29, 0.09)	0.365	-0.005	(-0.015, 0.005)	0.292
model 3		Reference	-0.07	(-0.27, 0.12)	-0.09	(-0.28, 0.10)	0.418	-0.006	(-0.016, 0.004)	0.263
**DST-b**	901									
crude model		Reference	0.17	(-0.11, 0.45)	0.10	(-0.17, 0.36)	0.951	0.000	(-0.012, 0.013)	0.946
model 1		Reference	0.07	(-0.16, 0.30)	0.02	(-0.19, 0.24)	0.835	-0.001	(-0.011, 0.010)	0.898
model 2		Reference	0.04	(-0.17, 0.26)	0.00	(-0.20, 0.21)	0.740	-0.002	(-0.013, 0.008)	0.674
model 3		Reference	0.05	(-0.17, 0.26)	-0.02	(-0.22, 0.19)	0.518	-0.005	(-0.015, 0.006)	0.402

Multivariable linear regression was used to assess longitudinal association to compare the 2-year changes in cognitive function across categories of hydration status, as well as continuous associations between cognitive function variables and hydration status.

Multivariable model 1: adjusted for baseline corresponding cognitive function score, age (years), sex, intervention PREDIMED-Plus randomized group, and participating center (≤100, 100 to <150, 150 to <200, >200 participants).

Multivariable model 2: additionally adjusted for body mass index (kg/m^2), educational level (primary, secondary or college), civil status (single, divorced or separated; married; widower), smoking status (current, former, or never), and physical activity (METs/min/day).

Multivariable model 3: additionally adjusted for sleep status (hours per day), depressive symptomatology (yes/no), diabetes prevalence (yes/no), hypertension (yes/no), hypercholesterolemia (yes/no), energy intake (kcal/day), alcohol consumption in g/day (and adding the quadratic term), and caffeine intake (mg/day).

[a]SOSM represents calculated serum osmolarity, and was calculated using the formula SOSM = 1.86 × (Na+ + K+) + 1.15 × glucose + BUN + 14, where all analytes are in mmol/L.

SOSM >300 mmol/L represented dehydration, 295 to 300 mmol/L represented impending dehydration, and <294 mmol/L represented hydration.

[b]GCF represents global cognitive function, and was calculated using a composite of z-scores in the formula: GCF=(zMMSE + zCDT + zVFT-a + zVFT-b + (–zTMT-A) + (–zTMT-B) + zDST-f + zDST-b) / 8.

Where MMSE, CDT, VFT-a, VFT-p, TMT-A, TMT-B, DST-f, and DST-b are various cognitive function tests that were included in the battery of neuropsychological tests.

Abbreviations: CDT, Clock Drawing Test; DST-b, Digit Span test-backward; DST-f, Digit Span test -forward; GCF, Global Cognitive Function; MMSE, Mini-Mental State Examination; TMT-A, Trail Making Test Part A; TMT-B, Trail Making Test Part B; VFT-a, Verbal Fluency tasks semantical; VFT-p, Verbal Fluency tasks phonological.

Supplemental Table 5. Sensitivity analysis of the multivariable adjusted mean 2-year change (β-coefficients and 95% CIs) in global cognitive function (GCF[a], n=1395) according to water and fluid intake related exposures in the PREDIMED-Plus cohort.

**WATER INTAKE (BEVERAGE)[b]**											
	Categorical								Continuous[c]		
Intake Range (ml/day)	<500	500 to 1000		1000 to 1500		>1500		p for trend			
	β (95% CI)	β	(95% CI)	β	(95% CI)	β	(95% CI)		β	(95% CI)	p-value
Model 3 + eGFR[d]	Reference	0.01	(-0.07, 0.09)	0.00	(-0.09, 0.08)	-0.01	(-0.11, 0.09)	0.761	-0.00001	(-0.00007, 0.00005)	0.805
Model 3 + Dietary Factors[e]	Reference	-0.07	(-0.21, 0.08)	-0.07	(-0.23, 0.09)	-0.01	(-0.18, 0.16)	0.863	0.00000	(-0.00011, 0.00010)	0.963
Model 3 + MMSE<24[f]	Reference	0.08	(-0.03, 0.18)	0.06	(-0.05, 0.17)	-0.04	(-0.17, 0.08)	0.555	-0.00002	(-0.00010, 0.00006)	0.568
Model 3 + GCF [<5 and >95%][g]	Reference	0.08	(-0.03, 0.18)	0.06	(-0.05, 0.17)	-0.04	(-0.17, 0.08)	0.555	-0.00002	(-0.00010, 0.00006)	0.568
**WATER FROM ALL FLUIDS[h]**											
	Quantiles of Water from All Fluids								Continuous[c]		
	Q1	Q2		Q3		Q4		p for trend			
	β (95% CI)	β	(95% CI)	β	(95% CI)	β	(95% CI)		β	(95% CI)	p-value
Model 3 + eGFR[d]	Reference	-0.03	(-0.11, 0.06)	-0.04	(-0.13, 0.05)	0.00	(-0.08, 0.08)	0.980	-0.00001	(-0.00006, 0.00004)	0.648
Model 3 + Dietary Factors[e]	Reference	-0.06	(-0.17, 0.05)	-0.04	(-0.15, 0.07)	-0.03	(-0.15, 0.08)	0.642	-0.00003	(-0.00009, 0.00004)	0.383
Model 3 + MMSE<24[f]	Reference	0.07	(-0.03, 0.17)	0.02	(-0.09, 0.13)	0.00	(-0.11, 0.10)	0.731	0.00000	(-0.00006, 0.00007)	0.924
Model 3 + GCF [<5 and >95%][g]	Reference	-0.01	(-0.13, 0.10)	-0.03	(-0.15, 0.09)	0.03	(-0.09, 0.15)	0.636	0.00000	(-0.00008, 0.00007)	0.955
**TOTAL WATER[i]**											
	Quantiles of Total Water Intake								Continuous[c]		
	Q1	Q2		Q3		Q4		p for trend			
	β (95% CI)	β	(95% CI)	β	(95% CI)	β	(95% CI)		β	(95% CI)	p-value
Model 3 + eGFR[d]	Reference	0.01	(-0.07, 0.09)	-0.06	(-0.15, 0.03)	-0.02	(-0.10, 0.07)	0.439	-0.00002	(-0.00006, 0.00002)	0.382
Model 3 + Dietary Factors[e]	Reference	-0.05	(-0.16, 0.06)	-0.03	(-0.14, 0.08)	-0.06	(-0.17, 0.05)	0.392	-0.00003	(-0.00009, 0.00002)	0.251
Model 3 + MMSE<24[f]	Reference	0.07	(-0.06, 0.20)	-0.03	(-0.16, 0.10)	-0.01	(-0.14, 0.12)	0.600	-0.00001	(-0.00008, 0.00006)	0.686
Model 3 + GCF [<5 and >95%][g]	Reference	0.07	(-0.06, 0.20)	-0.03	(-0.16, 0.10)	-0.01	(-0.14, 0.12)	0.600	-0.00001	(-0.00008, 0.00006)	0.686

Multivariable linear regression was used to assess longitudinal association to compare the 2-year changes in cognitive function across categories of hydration status and various modes of water/fluid intake, as well as continuous associations between cognitive function variables and hydration status/fluid intake.

Multivariable model 3: adjusted for baseline corresponding cognitive function score, age (years), sex, intervention PREDIMED-Plus randomized group, participating center (≤100, 100 to <200, 200 to <300, >300 participants), body mass index (kg/m2), educational level (primary, secondary or college), civil status (single, divorced or separated, married, widower), smoking habit (current, former, or never), and physical activity (METs/min/day), sleep status (hours per day), depressive symptomatology (yes/no), diabetes prevalence (yes/no), hypertension (yes/no), hypercholesterolemia (yes/no), energy intake (kcal/day), alcohol consumption in g/day (and adding the quadratic term), and caffeine intake (mg/day).

[a]GCF represents global cognitive function, and was calculated using a composite of z-scores using the formula: GCF=(zMMSE + zCDT + zVFT-a + zVFT-b + (–zTMT-A) + (–zTMT-B) + zDST-f + zDST-b) / 8.

Where MMSE, CDT, VFT-a, VFT-p, TMT-A, TMT-B, DST-f, and DST-b are various cognitive function tests that were included in the battery of neuropsychological tests.

[b]Water Intake represents fluid water consumed as a beverage, including tap and bottled forms of water.

[c]Continuous analysis based on 100 ml increments of water intake from the noted sources.

[d]eGFR represents estimated glomerular filtration rate, an indicator of renal function derived based on serum creatinine level, age and sex.

[e]Dietary factors represent dietary intake covariates (amount [g/day] of vegetables, fruit, legumes, grains, non-fluid dairy, meat, oils, fish, nuts, and pastries determined via the validated 143-item semi-quantitative Food Frequency Questionnaire (FFQ).

[f]MMSE <24 refers to the removal of participants with baseline MMSE values <24.

[g]GCF [<5% and >95%] refers to the removal of participants with extreme GCF z-scores at baseline, specifically <5% and >95%.

[h]Water from all fluids represents water consumed from all beverages.

[i]Total Water represents water consumed via beverage and food sources.

Abbreviations: CDT, Clock Drawing Test; DST-b, Digit Span test-backward; DST-f, Digit Span test -forward; eGFR, estimated Glomerular Filtration Rate; GCF, Global Cognitive Function; MMSE, Mini-Mental State Examination; Model 3, Multivariable Model 3 (defined above); Q, quantile; TMT-A, Trail Making Test Part A; TMT-B, Trail Making Test Part B; VFT-a, Verbal Fluency tasks semantical; VFT-p, Verbal Fluency tasks phonological.

Supplemental Table 6. Sensitivity analysis of the multivariable adjusted mean 2-year change (β-coefficients and 95% CIs) in global cognitive function (GCF[a], n=1395) according to EFSA fluid intake related guidelines in the PREDIMED-Plus cohort.

**EFSA GUIDANCE[b] FOR TOTAL FLUID WATER INTAKE (EFSA TFWI)**				
Total Fluid Water Intake	Does Not Meet Recommendations β (95% CI)	Meets Recommendations β	(95% CI)	p for trend
Model 3 + eGFR[d]	Reference	-0.03	(-0.09, 0.03)	0.313
Model 3 + Dietary Factors[d]	Reference	-0.01	(-0.09, 0.03)	0.643
Model 3 + MMSE<24[e]	Reference	-0.02	(-0.08, 0.04)	0.449
Model 3 + GCF [<5 and >95%][f]	Reference	-0.02	(-0.08, 0.04)	0.577

**EFSA GUIDANCE[b] FOR TOTAL WATER INTAKE (EFSA TWI)**				
Total Water Intake	Does Not Meet Recommendations β (95% CI)	Meets Recommendations β	(95% CI)	p for trend
Model 3 + eGFR[d]	Reference	-0.08	(-0.15, 0.00)	**0.047**
Model 3 + Dietary Factors[d]	Reference	-0.06	(-0.14, 0.02)	0.116
Model 3 + MMSE<24[e]	Reference	-0.06	(-0.14, 0.01)	0.111
Model 3 + GCF [<5 and >95%][f]	Reference	-0.07	(-0.15, 0.01)	0.068

Multivariable linear regression was used to assess longitudinal association to compare the 2-year changes in cognitive function across categories of hydration status and various modes of water/fluid intake, as well as continuous associations between cognitive function variables and hydration status/fluid intake.

Multivariable model 3: adjusted for baseline corresponding cognitive function score, age (years), sex, intervention PREDIMED-Plus randomized group, participating center (≤100, 100 to <200, 200 to <300, >300 participants), for body mass index (kg/m2), educational level (primary, secondary or college), civil status (single, divorced or separated, married, widower), smoking habit (current, former, or never), physical activity (METs/min/day), sleep status (hours per day), depressive symptomatology (yes/no), diabetes prevalence (yes/no), hypertension (yes/no), hypercholesterolemia (yes/no), energy intake (kcal/day), alcohol consumption in g/day (and adding the quadratic term), and caffeine intake (mg/day).

[a]GCF represents global cognitive function, and was calculated using a composite of z-scores using the formula: GCF=(zMMSE + zCDT + zVFT-a + zVFT-b + (–zTMT-A) + (–zTMT-B) + zDST-f + zDST-b) / 8.

Where MMSE, CDT, VFT-a, VFT-p, TMT-A, TMT-B, DST-f, and DST-b are various cognitive function tests that were included in the battery of neuropsychological tests.

[b]Based on the regulations and guidance of the EFSA Panel on Dietetic Products, Nutrition, and Allergies (NDA): Scientific Opinion on Dietary reference values for water. Where EFSA TFWI refers to the recommended levels of total fluid water intake for older adults at 2.0 L/day and 1.6 L/day for men and women, respectively, and EFSA TWI refers to the recommended levels of total water intake, from fluids and foods, for older adults at 2.5 L/day and 2.0 L/day for men and women, respectively.

Abbreviations: CDT, Clock Drawing Test; DST-b, Digit Span test-backward; DST-f, Digit Span test -forward; EFSA, European Food Safety Authority; GCF, Global Cognitive Function; MMSE, Mini-Mental State Examination; Multivariable Model 3 (defined above); Q, quantile; TMT-A, Trail Making Test Part A; TMT-B, Trail Making Test Part B; VFT-a, Verbal Fluency tasks semantical; VFT-p, Verbal Fluency tasks phonological.

Supplemental Table 7. Sensitivity analysis of the multivariable adjusted mean 2-year change (β-coefficients and 95% CIs) in global cognitive function (GCF[a]) according to hydration status in the PREDIMED-Plus cohort.

HYDRATION STATUS (SOSM[b])										
		Categorical						Continuous		
**SOSM (mmol/L, range)**		263 to 294	295 to 299		≥300					
	N	β (95% CI)	β	(95% CI)	β	(95% CI)	p for trend	β	(95% CI)	p-value
Model 3 + eGFR[c]	873	Reference	-0.05	(-0.18, 0.08)	-0.10	(-0.23, 0.03)	0.079	-0.010	(-0.017, -0.003)	**0.003**
Model 3 + Dietary Factors[d]	875	Reference	-0.07	(-0.21, 0.06)	-0.12	(-0.26, 0.01)	**0.048**	-0.011	(-0.018, -0.004)	**0.001**
Model 3 + MMSE<24[e]	849	Reference	-0.07	(-0.20, 0.06)	-0.12	(-0.25, 0.01)	**0.046**	-0.010	(-0.017, 0.004)	**0.002**
Model 3 + GCF [<5 and >95%][f]	710	Reference	-0.03	(-0.17, 0.12)	-0.07	(-0.21, 0.08)	0.235	-0.009	(-0.017, 0.001)	**0.025**

Multivariable linear regression was used to assess longitudinal association to compare the 2-year changes in cognitive function across categories of hydration status, as well as continuous associations between cognitive function variables and hydration status.

Multivariable model 3: adjusted for baseline corresponding cognitive function score, age (years), sex, intervention PREDIMED-Plus randomized group, participating center (≤100, 100 to <150, 150 to <200, >200 participants), body mass index (kg/m^2), educational level (primary, secondary or college), civil status (single, divorced or separated; married; widower), smoking status (current, former, or never), physical activity (METs/min/day), sleep status (hours per day), depressive symptomatology (yes/no), diabetes prevalence (yes/no), hypertension (yes/no), hypercholesterolemia (yes/no), energy intake (kcal/day), alcohol consumption in g/day (and adding the quadratic term), and caffeine intake (mg/day).

[a]GCF represents global cognitive function, and was calculated using a composite of z-scores using the formula: GCF=(zMMSE + zCDT + zVFT-a + zVFT-b + (–zTMT-A) + (–zTMT-B) + zDST-f + zDST-b) / 8.

Where MMSE, CDT, VFT-a, VFT-p, TMT-A, TMT-B, DST-f, and DST-b are various cognitive function tests that were included in the battery of neuropsychological tests.

[b]SOSM represents calculated serum osmolarity, and was calculated using the formula SOSM = 1.86 × (Na^+ + K^+) + 1.15 × glucose + BUN + 14, where all analytes are in mmol/L.

[c]eGFR represents estimated glomerular filtration rate, an indicator of renal function derived based on serum creatinine level, age, and sex.

[d]Dietary factors represent dietary intake covariates (amount [g/day] of vegetables, fruit, legumes, grains, non-fluid dairy, meat, oils, fish, nuts, and pastries determined via the validated 143-item semi-quantitative Food Frequency Questionnaire (FFQ).

[e]MMSE <24 refers to the removal of participants with baseline MMSE values <24.

[f]GCF [<5% and >95%] refers to the removal of participants with extreme GCF z-scores at baseline, specifically <5% and >95%.

Abbreviations: CDT, Clock Drawing Test; DST-b, Digit Span test-backward; DST-f, Digit Span test -forward; eGFR, estimated Glomerular Filtration Rate; GCF, Global Cognitive Function; MMSE, Mini-Mental State Examination; Model 3, Multivariable Model 3 (defined above); Q, quantile; TMT-A, Trail Making Test Part A; TMT-B, Trail Making Test Part B; VFT-a, Verbal Fluency tasks semantical; VFT-p, Verbal Fluency tasks phonological.

FIGURES

Figure 1. Flow diagram of participants in the PREDIMED-Plus sub-study for the analysis of a priori water intake and hydration status and cognitive performance in the PREDIMED-Plus trial.

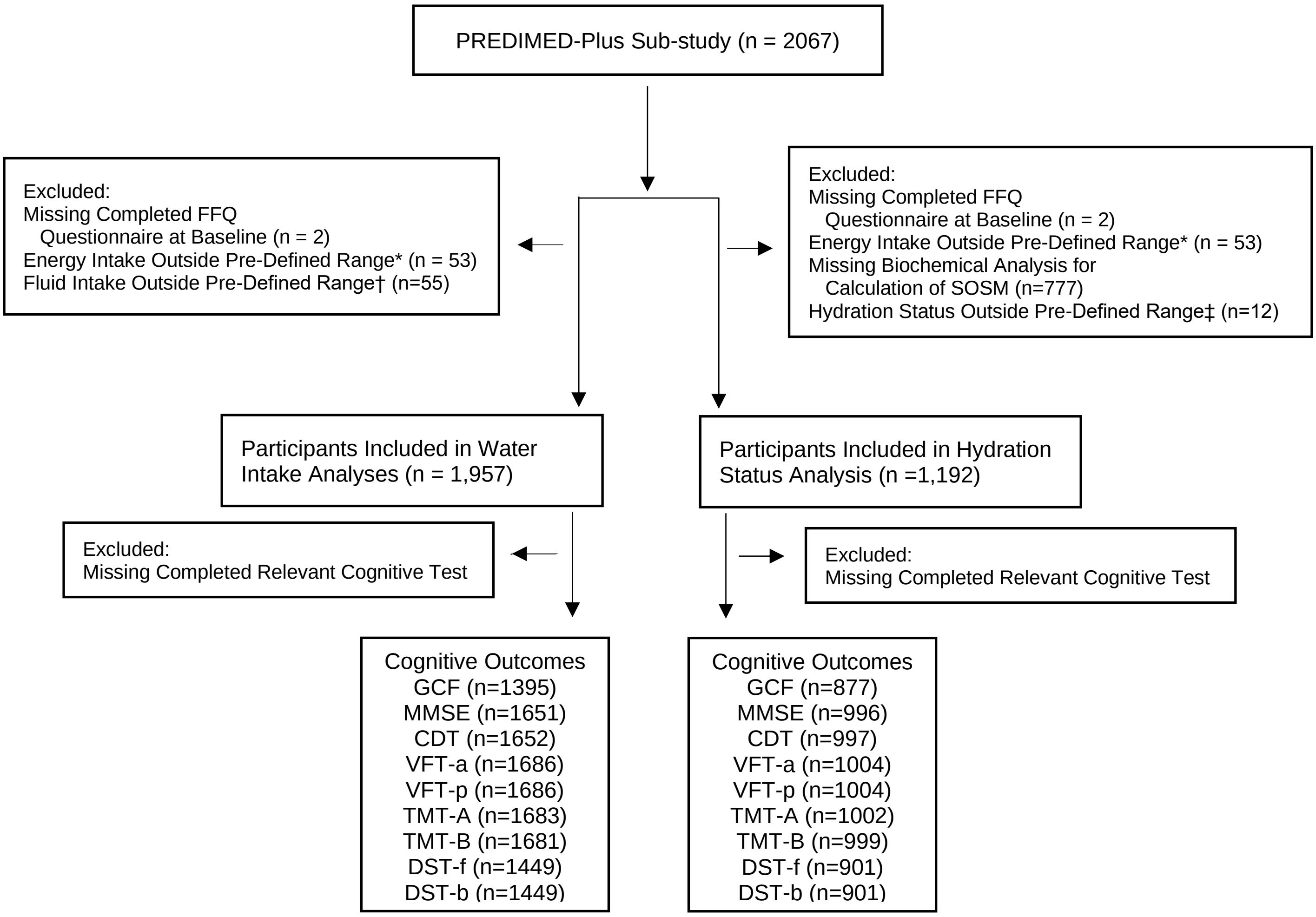

* Energy intakes outside pre-specified limits were defined as ≤800 kcal/d or ≥4000 kcal/d for men and ≤500 kcal/d or ≥3500 kcal/d for women.

†Fluid intakes outside pre-specified limits were defined as <490 ml/day or >3262 ml/day for men and <397 ml/day or >3590 ml/day for women or considered “outliers” (using the interquartile range method, by sex).

‡Hydration status outside pre-specified limits were defined as calculated serum osmolarity below <100 mmol/L.

Abbreviations: FFQ, food frequency questionnaire; SOSM, serum osmolarity.

Figure 2. Beta-coefficients and 95% CI for hydration status and water and fluid intakes continuously with 2-year changes in global cognitive function (z-scores) in women (A) and men (B).

A) Women

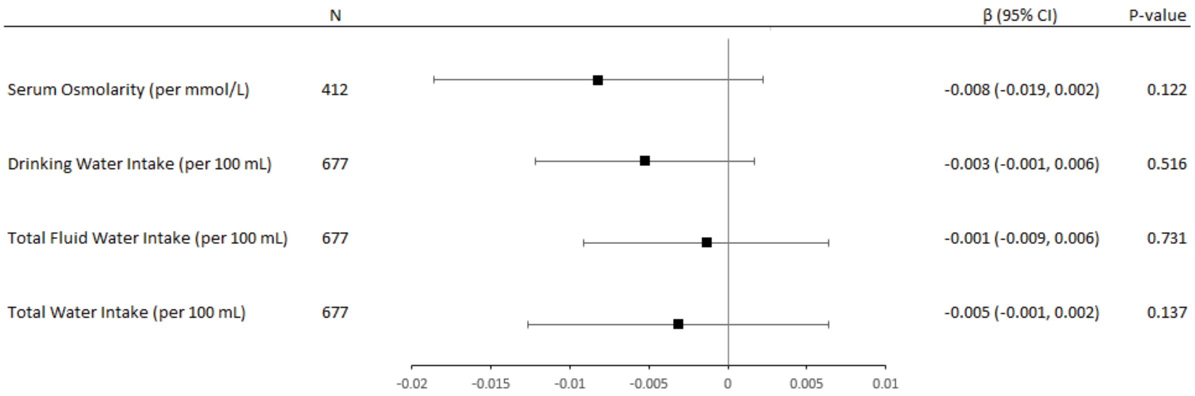

B) Men

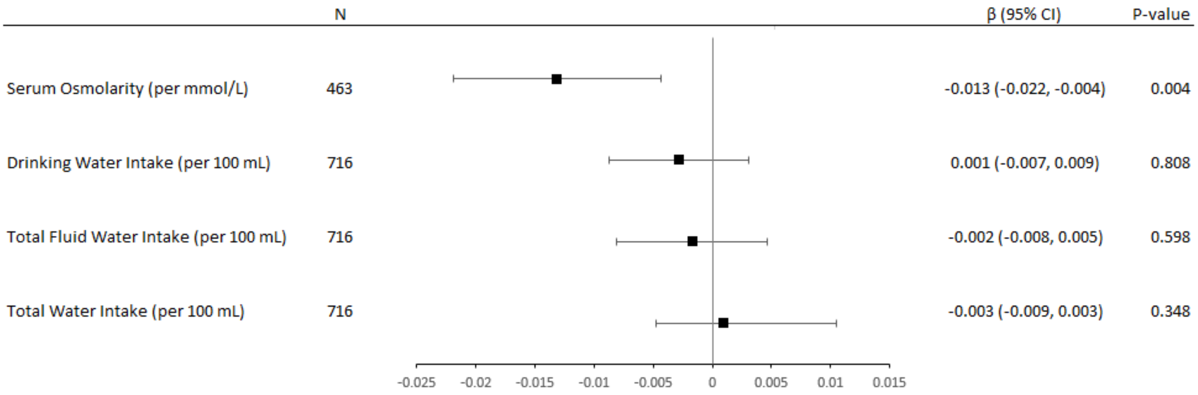

Linear regression models were adjusted for baseline covariates, including: baseline GCF score, age (years), sex, intervention PREDIMED-Plus randomized group, and participating center (≤100, 100 to <150, 150 to <200, >200 participants), body mass index (kg/m^2), educational level (primary, secondary or college), civil status (single, divorced or separated, married, widower), smoking habit (current, former, or never), and physical activity (METs/min/day), sleep status (hours per day), depressive symptomatology (yes/no), diabetes prevalence (yes/no), hypertension (yes/no), hypercholesterolemia (yes/no), energy intake (kcal/day), alcohol consumption in g/day (and adding the quadratic term), and caffeine intake (mg/day).

Beta represents changes in global cognitive function, expressed as z-scores, with each hydration or fluid intake component continuously.

Figure 3. Beta-coefficients and 95% CI for hydration status and water and fluid intakes categorically with 2-year changes in global cognitive function (z-scores) in women (A) and men (B).

A) Women

	N	β (95% CI)	P-value
Hydration Status[a]			
SOSM ≥300 mmol/L	245	-0.11 (-0.24, 0.02)	0.058
SOSM 295 to 300 mmol/L	134	-0.06 (-0.19, 0.08)	
SOSM <295 mmol/L	33	Reference	
Drinking Water[b]			
>1500 mL/d	99	-0.01 (-0.11, 0.09)	0.761
1000-1500 mL/d	213	-0.003 (-0.09, 0.08)	
500-1000 mL/d	244	0.01 (-0.07, 0.09)	
≤500 mL/d	121	Reference	
Water, All Fluids[c]			
Q4: >2225 mL/d	169	-0.005 (-0.09, 0.08)	0.87
Q3: 1810-2222 mL/d	169	-0.05 (-0.13, 0.04)	
Q2: 1445-1809 mL/d	168	-0.02 (-0.11, 0.06)	
Q1: ≤1443 mL/d	171	Reference	
Total Water[d]			
Q4: >3308 mL/d	173	-0.05 (-0.14, 0.04)	0.137
Q3: 2832-3307 mL/d	174	-0.07 (-0.16, 0.02)	
Q2: 2396-2832 mL/d	161	0.003 (-0.08, 0.09)	
Q1: <2395 mL/d	169	Reference	
EFSA TFWI[e]			
Meets Recommendation	436	-0.06 (-0.14, 0.02)	0.116
Does Not Meet Recommendation	241	Reference	
EFSA TWI[f]			
Meets Recommendation	620	-0.03 (-0.09, 0.03)	0.375
Does Not Meet Recommendation	57	Reference	

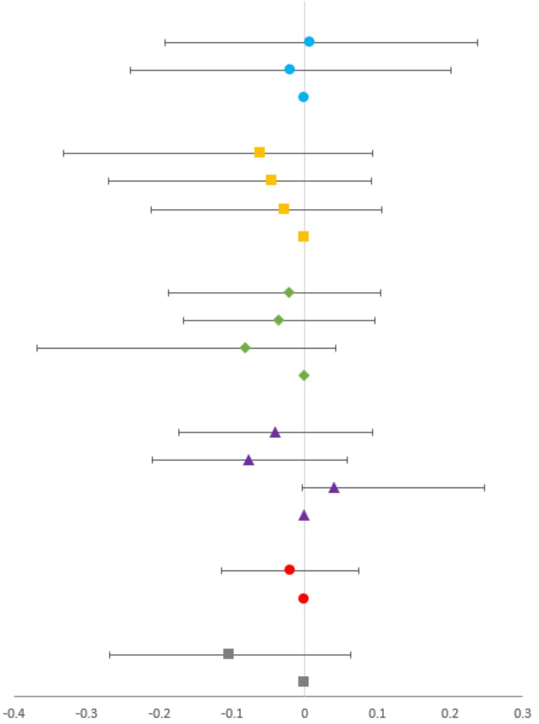

B) Men

	N	β (95% CI)	P-value
Hydration Status[a]			
SOSM ≥300 mmol/L	245	-0.20 (-0.36, -0.04)	0.002
SOSM 295 to 300 mmol/L	169	-0.07 (-0.23, 0.10)	
SOSM <295 mmol/L	51	Reference	
Drinking Water[b]			
>1500 mL/d	111	0.03 (-0.10, 0.16)	0.711
1000-1500 mL/d	199	0.03 (-0.08, 0.15)	
500-1000 mL/d	270	0.05 (-0.06, 0.16)	
≤500 mL/d	138	Reference	
Water, All Fluids[c]			
Q4: >2225 mL/d	206	0.02 (-0.09, 0.13)	0.906
Q3: 1810-2222 mL/d	187	-0.05 (-0.17, 0.06)	
Q2: 1445-1809 mL/d	164	0.05 (-0.06, 0.16)	
Q1: ≤1443 mL/d	161	Reference	
Total Water[d]			
Q4: >3308 mL/d	201	-0.06 (-0.18, 0.06)	0.302
Q3: 2832-3307 mL/d	184	-0.05 (-0.17, 0.06)	
Q2: 2396-2832 mL/d	173	-0.03 (-0.14, 0.08)	
Q1: <2395 mL/d	160	Reference	
EFSA TFWI[e]			
Meets Recommendation	311	-0.03 (-0.11, 0.05)	0.507
Does Not Meet Recommendation	407	Reference	
EFSA TWI[f]			
Meets Recommendation	509	-0.05 (-0.14, 0.04)	0.262
Does Not Meet Recommendation	209	Reference	

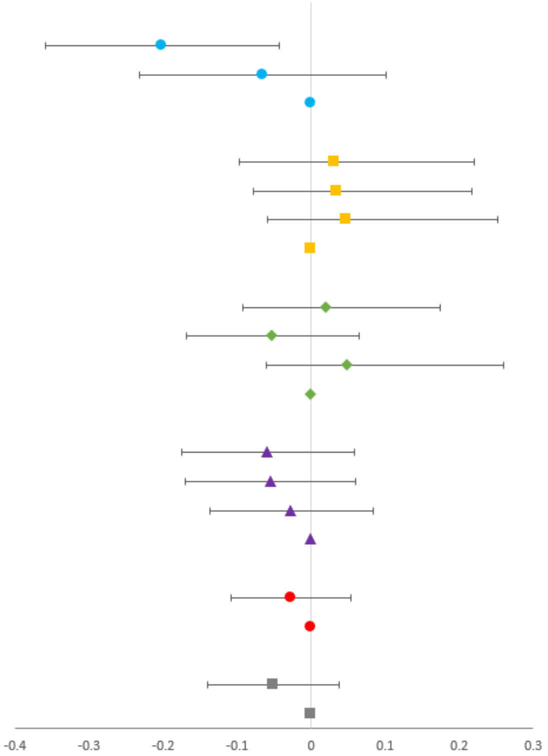

Linear regression models were adjusted for baseline covariates, including: baseline GCF score, age (years), intervention PREDIMED-Plus randomized group, and participating center (for hydration status: ≤100, 100 to <150, 150 to <200, >200 participants; for fluid-related exposures: ≤100, 100 to <200, 200 to <300, >300 participants), body mass index (kg/m^2), educational level (primary, secondary or college), civil status (single, divorced or separated, married, widower), smoking habit (current, former, or never), and physical activity (METs/min/day), sleep status (hours per day), depressive symptomatology (yes/no), diabetes prevalence (yes/no), hypertension (yes/no), hypercholesterolemia (yes/no), energy intake (kcal/day), alcohol consumption in g/day (and adding the quadratic term), and caffeine intake (mg/day).

[a]Hydration Status refers to serum osmolarity, where dehydration, impending dehydration, and hydrated statuses were defined as SOSM >300, 295-300, and <295 mmol/L, respectively

[b]Drinking Water refers to tap and bottled water intakes.

[c]Water, All Fluids refers to tap and bottled water, plus water from other beverages and fluid food sources, such as soups and smoothies.

[d]Total Water refers to water from all fluids in addition to water present in food sources.

[e]EFSA TFWI refers to the recommended levels of total fluid water intake for older adults at 2.0 L/day and 1.6 L/day for men and women, respectively.

[f]EFSA TWI refers to the recommended levels of total water intake, from fluids and foods, for older adults at 2.5 L/day and 2.0 L/day for men and women, respectively.

Abbreviations: EFSA, European Food Safety Authority; TFWI, total fluid water intake; TWI, total water intake; SOSM, serum osmolarity.
